# Improved emotion differentiation under reduced acoustic variability of speech in autism

**DOI:** 10.1186/s12916-024-03341-y

**Published:** 2024-03-14

**Authors:** Mathilde Marie Duville, Luz María Alonso-Valerdi, David I. Ibarra-Zarate

**Affiliations:** Escuela de Ingeniería y Ciencias, Tecnologico de Monterrey, Ave. Eugenio Garza Sada 2501 Sur, Col: Tecnológico, Monterrey, N.L 64700 México

**Keywords:** Autism, Emotion, Prosody, Pediatrics, Naturalness, Speech, Voice, Sensory variability, Electroencephalography

## Abstract

**Background:**

Socio-emotional impairments are among the diagnostic criteria for autism spectrum disorder (ASD), but the actual knowledge has substantiated both altered and intact emotional prosodies recognition. Here, a Bayesian framework of perception is considered suggesting that the oversampling of sensory evidence would impair perception within highly variable environments. However, reliable hierarchical structures for spectral and temporal cues would foster emotion discrimination by autistics.

**Methods:**

Event-related spectral perturbations (ERSP) extracted from electroencephalographic (EEG) data indexed the perception of anger, disgust, fear, happiness, neutral, and sadness prosodies while listening to speech uttered by (a) human or (b) synthesized voices characterized by reduced volatility and variability of acoustic environments. The assessment of mechanisms for perception was extended to the visual domain by analyzing the behavioral accuracy within a non-social task in which dynamics of precision weighting between bottom-up evidence and top-down inferences were emphasized. Eighty children (mean 9.7 years old; standard deviation 1.8) volunteered including 40 autistics. The symptomatology was assessed at the time of the study via the Autism Diagnostic Observation Schedule, Second Edition, and parents’ responses on the Autism Spectrum Rating Scales. A mixed within-between analysis of variance was conducted to assess the effects of group (autism versus typical development), voice, emotions, and interaction between factors. A Bayesian analysis was implemented to quantify the evidence in favor of the null hypothesis in case of non-significance. Post hoc comparisons were corrected for multiple testing.

**Results:**

Autistic children presented impaired emotion differentiation while listening to speech uttered by human voices, which was improved when the acoustic volatility and variability of voices were reduced. Divergent neural patterns were observed from neurotypicals to autistics, emphasizing different mechanisms for perception. Accordingly, behavioral measurements on the visual task were consistent with the over-precision ascribed to the environmental variability (sensory processing) that weakened performance. Unlike autistic children, neurotypicals could differentiate emotions induced by all voices.

**Conclusions:**

This study outlines behavioral and neurophysiological mechanisms that underpin responses to sensory variability. Neurobiological insights into the processing of emotional prosodies emphasized the potential of acoustically modified emotional prosodies to improve emotion differentiation by autistics.

**Trial registration:**

BioMed Central ISRCTN Registry, ISRCTN18117434. Registered on September 20, 2020.

**Supplementary Information:**

The online version contains supplementary material available at 10.1186/s12916-024-03341-y.

## Background

Understanding speakers’ emotional states conveyed by speech prosody requires decoding relevant acoustic features in voice and relating them to previously learned social and cognitive representations. Autism spectrum disorder (ASD) is distinguished by altered emotional speech perception that has been observed both at behavioral and neurophysiological scales. Perception may be challenged by (a) the generation of causal inferences about the sensory evidence and (b) the confidence placed towards the sensory information relative to prior expectations. Imagine that you are listening to an audiobook telling a hectic introspective scenario. However, your reading system is broken so that syllables are shuffled creating pseudowords from which you cannot understand the meaning. If prosodic contours still match your prior knowledge about emotional and social patterns (expectations), then you could infer some semantic aspects of the story. Particularly, your perception would be weighted towards prior knowledge, and you would sample the sensory environment to maximize the likelihood between expectations and sensory cues. Nevertheless, if prosodic patterns are unexpected and ambiguous, your perception would be weighted towards more fine-grained sensory sampling. You would learn new probabilistic structures of acoustic cues to update prior expectations and infer meaning. In sum, emotion perception starts from the weighted decoding of sensory cues to promote later in-depth appraisal of affective states. In this context, the autistic affective perception may be substantiated by unusual weighting dynamics, leading to perceptual inferences less sensitive to abstract socio-cognitive representations [1].

Recent findings highlighted lower accuracy at recognizing discrete emotional categories by autistic children (e.g., differentiate between sadness, happiness, and neutral prosodies [2]). Besides, the performance may be worsened by increasing the complexity of the social context (e.g., for complex emotions and most ecological social scenarios [3]). The perception of emotional prosodies by autistics has been substantiated by atypical neural activity linking sensory to higher-order processing. For instance, the hyperconnectivity with the temporo-parietal junction may outline aberrant signalization of the sensory processing for mental state inferences [2]. However, the hypoconnectivity between higher-order regions (e.g., the superior temporal sulcus and the amygdala) may underpin weakened socio-emotional inferences [3]. Furthermore, lower verbal intelligence may be a non-negligible covariate to explain emotion identification deficits. Particularly, it refers to verbal labeling into abstract concepts (prior knowledge, e.g., identifying a sensation within a conceptual dimension such as discrete labeling, valence, or arousal). [4]. As such, impairments have been associated with more effortful cognitive processing during explicit tasks of emotion identification, indexed by higher amplitude of event-related late potentials (400–1000 ms) extracted from electroencephalographic (EEG) data [4]. However, during passive listening, lower amplitude may outline weakened motivational and contextual inferences [5]. Thus, emotional impairments may be primarily fostered by perceptions estranged from high-order abstract inferences. Indeed, higher psychoacoustic abilities (i.e., low-level acoustic feature processing) and higher autistic symptomatology were associated with lower affective prosody recognition in speech. Nevertheless, at the highest autistic severity, increased access to fine-grained acoustic features may provide a compensatory mechanism to improve emotion categorization, although not sufficient to vanish impairments [6, 7]. In sum, the evidence from emotion perception in ASD outlined the unbalanced precision-weighting between sensory processing and prior knowledge towards perceptions closer to the acoustic characteristics of stimuli.

Contradictory findings also outlined no impairment for recognizing emotional prosody in speech at behavioral [8] or neural level [9] by autistics. Interestingly, emotion recognition and processing may depend on the acoustic and social complexity of stimuli and the experimental task, and results may differ according to behavioral or neurophysiological markers. For instance, the identification of basic emotions that are more explicit (e.g., anger, happiness) than complex emotions (e.g., jealousy, boredom) may rely on lower abstract decoding and precision to socio-cognitive priors to reach emotional saliency, therefore improving their accurate perception to autistics [10]. Also, the lexical complexity of affective speech (e.g., vowel, word, or sentence) and the implicit/explicit allocation to emotional information may mediate perceptive mechanisms according to the socio-cognitive intricacy induced by the stimulus for integrative processing. Additionally, the intensity of emotional vocalization may trigger differential sensory-prior precision balance for perception as high-intensity stimuli are characterized by the exaggeration of acoustic profiles of emotions that promote the reliability of sensory cues to recognize emotions, improving detection in ASD [4, 6] or favoring confusion between emotions that acoustically converge at higher intensity (e.g., happiness and anger) [11]. Besides, when the noisy information was reduced while listening to speech during clinical intervention designed to improve emotion recognition, autistics showed equal to better behavioral performance post-intervention. Neurophysiological data outlined more efficient emotion integration at early and late stages (reduced latency of EEG event-related potential) and emotion identification highlighted by significant amplitude differences of EEG components between emotions, although differential neurophysiological patterns between ASD and typically developed (TD) still emphasized divergent processing mechanisms [12]. Overall, the balance between processing sensory information and inferring emotion identification via internal socio-emotional representations may support autistic alterations for emotional prosody perception.

**Fig. 1:**
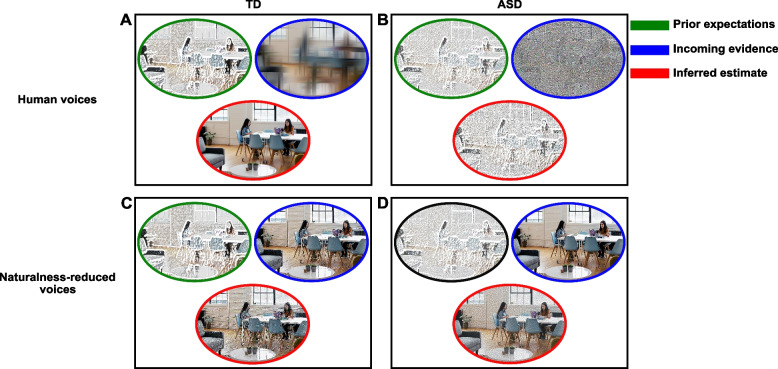
Precision-weighting for perception inferences during emotional speech. **A** Within highly unstable sensory environments, higher precision may be ascribed to socio-emotional priors while a coarse decoding of the sensory evidence may optimize the perception (inferred estimate) of emotional prosodies. **B** Autistic mechanisms of perception may overestimate the environmental variability (incoming evidence), and fine-grained bottom-up inputs would be misinterpreted as predictions, leading to blurry prosodic contours differentiation (inferred estimates). **C** The reduction of voice’s variability creates more stable and unknown environments that would promote the precision towards the incoming evidence by neurotypicals without jeopardizing the differentiation of emotional prosodies. **D** We hypothesize that more reliable sensory environments may favor the differentiation of emotional prosodies by autistics

Within this context, a Bayesian framework of perception may be considered. See Fig. 1 for a graphical representation. From this perspective, action and perception interact to minimize the expected surprise (i.e., prediction error or inverse likelihood). At neural levels, predictive coding suggests the constant generation of top-down inferences by higher-brain regions to build hidden-state hypotheses about the causes of sensory experiences and depict differences between sensory inputs and expectations (i.e., prediction error) [13]. In that respect, actions sample sensory inputs via controlling the precision (i.e., confidence for information) to conform predictions and maximize the likelihood [14]. Importantly, precision itself must be previously estimated towards the optimization of given goals. That is, the weighting of new evidence against prior beliefs must be dynamically adjusted according to context [15]. For instance, if you are listening to speech, trying to infer emotional states from prosodic contours, you may sample the most salient sensory information (e.g., loudness and speech rate). Thus, you would be increasing the informativeness (i.e., precision) of those acoustic features, relative to other irrelevant cues (e.g., frequency of harmonics), following your prior knowledge about emotion identification. You would also be decreasing the precision to the sensory variability relative to prior inferences. Therefore, perception is a constructive process by which information is weighted to optimize behavior relative to the degree of precision (or uncertainty) of both prior knowledge and sensory evidence. Particularly, when the prediction error is higher than the expected variability of inputs (e.g., cue outcome is unexpected based on the confidence previously ascribed to contextual volatility and variability), priors are updated to optimize internal modeling and solve future discordances [16]. That is, whenever the sampled sensory evidence does not match the top-down inferences (i.e., the cue is not predictive of the emotional prosody anymore), you would reevaluate the weighting between bottom-up and top-down information. Therefore, high sensory precision would increase the influence of bottom-up affordances by promoting the learning rate of contextual variability (i.e., the receptiveness to sensory variability and volatility), thus lessening the surprise to all inputs, and broadening the uncertainty of internal states. Contrarywise, higher precision to priors would decrease the expected variability of inputs, promote the learning of probabilistic distributions of variants, and bias perception towards internal representations [17, 18].

Precision should be mostly ascribed to priors whenever the sensory context is ambiguous (i.e., when fine-grained bottom-up information is not reliable). Nevertheless, the precision of priors about environmental changes (i.e., volatility and variability) must also be adjusted, so that the contextual stability is tracked to adjust future prediction errors. Particularly, moderately variable environments may entangle less precise priors and relatively increased confidence towards bottom-up inputs to optimize learning about new statistical patterns [18], whereas in highly unstable contexts, sensory information becomes extremely ambiguous so that precision should be mostly ascribed towards internal representations. For instance, affective speech encounters high temporal and spectral complexity where speaker, lexical, phonological, and emotional dependencies for perception may exist, creating highly equivocal sensory environments [19, 20]. In this case, the emotional perception would be mediated by coarse sensory cues decoding and higher confidence in internal knowledge of affective representations [21]. This mechanism is illustrated in Fig. 1A. In that sense, a multilayer framework for social cognition was proposed, where mental states (e.g., joyful) and individuals’ identities (e.g., optimistic) represent layers that are mapped onto low-dimensional spaces, and probabilistic between-dimension and within-layer connections define complex internal representations. In this context, only socially relevant sensory features that can be mapped onto state-trait dimensional spaces must be processed [22]. Thus, processing irrelevant fine-grained inputs may be disadvantageous for computing an accurate perception of emotional states.

Nevertheless, the autistic perception may be shaped by different dynamics of precision weighting, characterized by the overestimation of environmental variability and steady overweighting of sensory inputs wherein fine-grained bottom-up inputs would be misinterpreted as predictions (i.e., altered update of prior beliefs according to context) [23, 24]. See Fig. 1B for an illustration. Heterogeneity in uncertainty evaluation shapes individual and contextual differences in perception, since priors are built from subjective past experiences, and precision-weighting depends on environments and goals; however, it becomes atypical in ASD [25]. Recent findings also outlined a slow update of internal probabilistic environmental representations. That is, autistics may form typical prior beliefs, but the update rate may be slower, ascribing more precision to past versus recent inputs [26]. Other findings however highlighted intact flexibility of the learning rate towards environmental change [27, 28]. Therefore, precision weighting may be altered in autistics, but the presence and nature of atypicality may depend on tasks, contexts, and processed information [29]. However, the actual knowledge tends to evidence the over-precision to sensory variability in socio-emotional contexts [1, 30].

The present study aims to assess the perception of emotional prosody when conveyed by human or naturalness-reduced (i.e., less volatile, reduced variability) voices in ASD and TD. Naturalness-reduced voices were characterized by simplified acoustic environments (i.e., simpler hierarchical structures for spectral and temporal cues; for a detailed description of voices, see [31]). The emotion recognition conveyed by both human and naturalness-reduced voices was previously confirmed in TD [31, 32] (Fig. 1C), as well as the perception of the human voices by TD and autistic children, highlighting impaired neurophysiological markers for emotion identification in ASD [33]. We hypothesize however that naturalness-reduced voices would foster emotion discrimination by autistics promoting differentiation, as illustrated in Fig. 1D. These results would be in line with a recent finding that highlighted more precise sensory emotional representations of point-light faces (i.e., simplified depiction of human faces), and equally accurate emotion identification compared to TD, suggesting that at lower sensory complexity, autistics may develop alternative mechanisms based on fine-grained sensory information to accurately differentiate emotions [30]. Emotion differentiation by ASD fostered by a less variable sensory environment is expected; however, the assessment of specificities about sensory and cognitive evaluations for emotional prosody encoding is out of the scope of this study although highly encouraged in future works.

A task-irrelevant (implicit) emotion perception paradigm was designed, in which relevant stimuli were allowed to assess precision-weighting mechanisms in a non-social visual task by a behavioral marker (accuracy) while emotions acted as distractors. Importantly, the cognitive load of the relevant task was optimized to partially deplete top-down attentional control resources towards relevant features (in typical processing) and allow emotion differentiation, as previously evidenced in a study with the same task, emotional stimuli, and sample characteristics [33]. EEG data were recorded from which event-related spectral perturbations (ERSP) were extracted that provided neurophysiological markers for the modulation of ongoing neural activity induced by the processing of emotional prosodies.

### Methods

This study was registered within BioMed Central (ISRCTN18117434; last access on September 4, 2023) [34].

### Participants

**Fig. 2:**
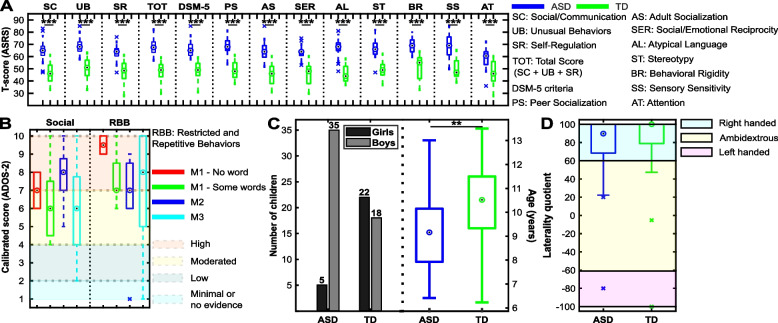
Children’s demographic and clinical characteristics. Autistic symptomatology measured by the **A** Autism Spectrum Rating Scales (ASRS, parents’ version), and the **B** Autism Diagnostic Observation Schedule, Second Edition (ADOS-2) at the time of the experiment; **C** gender and age; and **D** handedness. *M1* module 1; *M2* module 2; *M3* module 3. **: *p*-value < 0.01; ***: *p*-value < 0.001; *ASD* autistic; *TD* typically developed. Note that ASD and TD children differed as regards gender and age: see Additional file 1 and section Sensitivity analysis: age- and gender-controlled sample

Eighty children participated in the study (mean 9.7 years old; range [6.2–13.5]; standard deviation 1.8, 27 girls). All children were Mexican, currently living in Mexico, and have received Mexican familial and academic educations to align with the cultural shaping of emotional prosodies [32]. Standardized scores (*T*-scores) computed from answers by one parent on the Spanish version of the full-length Autism Spectrum Rating Scales (ASRS) [35] were gathered for every child at the time of the experiment and are presented in Fig. 2. *T*-scores equal to or higher than 60 indicate higher symptom severity than the one observed for the normative sample (TD children of the same age). Cronbach’s alpha index of internal reliability measured using R-4.3.1 [36] is presented in Table 1 for every scale of the ASRS (social/communication (SC), unusual behaviors (UB), self-regulation (SR), total score (TOT) (SC + UB + SR), Diagnostic and Statistical Manual of Mental Disorders-Fifth Edition (DSM-5) criteria, peer socialization (PS), adult socialization (AS), social/emotional reciprocity (SER), atypical language (AL), stereotypy (ST), behavioral rigidity (BR), sensory sensitivity (SS), attention (AT)) and group of participants (autistic: ASD, typically developed: TD). Coefficients confirm the reliability of the assessment by indicating a close relationship between the items of each scale.

**Table 1:** Cronbach’s alpha coefficients for each scale of the ASRS and group of participants

Scale	Number of items		ASD—no or few words	ASD—phrases or fluent	TD
	**No or few words**	**Phrases or fluent**			
SC	16	19	0.90	0.85	0.90
UB	18	24	0.91	0.91	0.82
SR	16	17	0.85	0.86	0.87
TOT	50	60	0.93	0.93	0.92
DSM-5	27	34	0.82	0.92	0.90
PS	7	9	0.93	0.80	0.78
AS	16	6	0.90	0.85	0.91
SER	12	13	0.90	0.82	0.85
AL	N/A	6	N/A	0.77	0.76
ST	5	5	0.74	0.76	0.76
BR	8	8	0.85	0.89	0.88
SS	6	6	0.86	0.82	0.78
AT	11	11	0.88	0.82	0.82

*ASD* autistic, *TD* typically developed

Half of the children had previously received a diagnosis of ASD by experienced clinicians (mean 9.2 years old; range [6.4–13]; standard deviation 1.7, 5 girls). Instruments reported by the parents were as follows: DSM-5 [37] (*n* = 29); Gilliam Autism Rating Scales, Third Edition [38] (*n* = 4); Childhood Autism Spectrum Test [39] (*n* = 2); Ángel Rivière’s autism spectrum inventory [40] (*n* = 3); Autism Diagnostic Interview–Revised [41] (*n* = 4); Autism Diagnostic Observation Schedule, Second Edition [42] (ADOS-2, *n* = 3); Mexican filter for Asperger’s detection [43] (*n* = 5); and Psychoeducational Profile, Third Edition [44] (*n* = 1). The diagnosis was confirmed for every child at the time of the experiment via ADOS-2 (module 1—no word: *n* = 2; module 1—some words: *n* = 8; module 2:*n* = 7; module 3:*n* = 23). The first author conducted the assessments, who meets the standard requirements for clinical administration. Calibrated scores and associated categories (minimal, low, moderated, high) that compare raw scores with the ones of other autistic children of the same age [45] are presented in Fig. 2.

The other half of the children were TD (mean 10.2 years old; range [6.2–13.5]; standard deviation 1.7, 22 girls). Within both groups, parents reported no history of psychiatric, cognitive, language, or hearing disorders (apart from ASD in the ASD group), and no child was under medication affecting the nervous system at the time of the study. They had normal or corrected-to-normal vision. Handedness was assessed before starting the experiment by the Spanish version of the Edinburgh Handedness Inventory [46]. Categories (left- and right-handed, ambidextrous) and laterality quotients are reported in Fig. 2.

R-4.3.1 [36] was used to test for age difference between the groups (ASD and TD) using Wilcoxon rank sum (Mann–Whitney) for unpaired samples. The level of significance was set at *p*-value < 0.05. Normality was assessed with the Shapiro–Wilk test (*W*_TD_ = 0.97, *p*-value_TD_ = 0.28; *W*_ASD_ = 0.94, *p*-value_ASD_ = 0.03) but was not confirmed for ASD, impeding the application of a two-sample *t*-test. No outlier was outlined (i.e., values out of 1.5 times the interquartile range). The Wilcoxon test indicated that TD children were older than ASD (*W* = 1082.5, *p*-value = 0.007, *r* = 0.304).

Also, a mixed within-between two-way analysis of variance (ANOVA) was conducted to assess the effect of group on *T-*scores: group × scale with “scale” as the repeated (within) factor and “group” as the independent (between) factor. A default of residuals’ normality was outlined by Shapiro–Wilk’s test (*W* = 0.99, *p*-value < 0.001). Therefore, a non-parametric approach was performed. Particularly, the aligned rank transform was used on the mixed effects. Then, the analysis of variance of aligned rank transformed data was conducted. From this analysis, a significant effect of group was highlighted: *F*(1,78) = 355.15, *p*-value < 0.001. Post hoc analyses were performed by Tukey’s procedure after applying a one-way ANOVA with the intersection between group and scale as a factor (Shapiro–Wilk test for normality of residuals: *W* = 1, *p*-value = 0.81). A violation of sphericity was highlighted by Mauchly’s test (*W* < 0.001, *p*-value < 0.001). Therefore, a Greenhouse–Geisser correction was conducted (*F*_GGe_(25,975) = 0.29, *p*-value < 0.001). Post hoc comparisons outlined higher scores for ASD children for every scale of the ASRS (*p*-value < 0.001) as represented in Fig. 2.

### Paradigm for task-irrelevant perception of emotional prosodies

The indirect perception of emotional prosodies was implemented to avoid biases from additional abstract verbal or graphical reasoning that may involve explicit tasks (e.g., choosing a word or emoji that corresponds to the prosody) [47, 48]. During the session, the child was seated in an armchair in front of a computer screen where a visual task (relevant) was displayed, while listening to the emotional prosodies (task-irrelevant). The EEG activity was recorded all-task long.

The visual task consisted of unique-target multiple object tracking (MOT). Namely, every MOT trial started with the 500-ms display of an empty gray square of 20 × 20° of visual angle on a black background. Then, four white discs (filled, diameter 1.3° of visual angle) appeared at random positions (but all different) inside the square and one disc (the target) immediately started flashing for 1s (the disc turned red four times with 200-ms intervals, remaining red for 400 ms the fourth time). The target became white again, and all discs started to follow random strait paths inside the frame for 8 s at 8° of visual angle (deg/s). The discs were reflected with the same angle as the incidence one whenever they touched an edge. They further remained still at their latest position until receiving the child’s response. The mouse pointer appeared on the screen, and the participant could select the target using the wireless mouse located on their dominant side. Disc selection immediately started the next trial. Whenever tracking was lost, the instruction was to guess and choose randomly. No feedback was received to avoid inducing emotions from the visual task. Therefore, the task required the sensory sampling of the most salient information (i.e., the target disc) following the prior knowledge of its informativeness to successfully answer the task.

The session was divided into three 40-trial MOT tasks and the child could relax the time needed between tasks. All tasks were equal (same 40-trial sequence) within and between children to avoid unwanted variance in performance from trial characteristics. Each task lasted approximately 8 min. Before starting the session, instructions were explained in writing and verbally, and the child was specified to ask all questions needed. Then, 3 practice trials were displayed (6 discs, including 1 target). The child was further indicated relaxing for 1 min to focus on the becoming task, during which the participant had to remain still with the hand on the mouse to reduce artifacts on EEG signals. MOT trials were created on PsychoPy3 (3.2.4) [49] after the edition of the open-source code shared by Meyerhoff and Papenmeier [50].

Stimuli for emotional prosodies were 144 Spanish single words (24 per emotion: anger, disgust, fear, happiness, neutral, and sadness) uttered by a Mexican female speaker extracted within the Mexican Emotional Speech Database (MESD) [31, 32, 51], available in Mendeley Data at http://doi.org/10.17632/cy34mh68j9.5. Words from the human voice and both levels of naturalness reduction (level 1 and level 2) were retrieved from MESD for a total of 432 utterances. Naturalness-reduced voices of MESD have decreased variability of acoustic components as regards pitch, formant frequency, lexical stress, harmonics intensity, and speech rate. Particularly, the frequency of the first and second formants and the amplitude of the second and fourth harmonics were reduced so that the spectral profile was simpler. Also, differences between stressed and unstressed syllables as regards pitch and duration were faded, so that the temporal complexity was decreased. Patterns for acoustic modulations were designed after outlining the acoustic components relevant to the perception of naturalness. The reduction of acoustic variability was progressive from level 1 (38%) to level 2 (74%). See [31] for a detailed explanation of their creation. The acoustic environment defines the emotional expression (anger, disgust, fear, happiness, neutral, or sadness) at all levels of naturalness (human, level 1, and level 2). For instance, happiness had a higher pitch than neutral prosodies (see [32] for a complete description of acoustic tendencies between emotions). Emotion identification based on acoustic features was previously ensured by implementing a supervised learning algorithm reaching 90.9% accuracy [32]. Accurate human discrimination of emotions was also emphasized by both adults ([31]) and children ([33]). Words were preferred over pseudowords to avoid biases from attentional and cognitive efforts to ignore the unknown structure of the words and ensure more ecological acoustic environments. Words’ frequency of use, familiarity, and concreteness did not differ between emotions, and written semantic aligned with the emotional prosody [32].

The Shure SRH1840 audio headset that has a flat frequency response was used to display the utterances at 70 dBA with a 3.11-s stimulus-onset asynchrony. Every type of voice was listened to while answering a 40-trial MOT task. Utterances were displayed randomly, and the order differed between but was the same within participants (i.e., each utterance was ranked equally within the sequence to its equivalent in the other types of voice) to avoid bias from order presentation. The order of presentation of human, level 1, and level 2 voices was randomly distributed between children. Utterances display and EEG recordings were implemented in OpenVibe (1.3.0) [52]. Finally, auditory stimuli acted as task-irrelevant distractors as participants were asked not to pay attention to them and to focus on the MOT task.

Note that the MOT task was previously designed to partially deplete top-down attentional resources and ensure the simultaneous processing of auditory stimuli by both autistics and neurotypicals [33]. Therefore, the performance on MOT would be influenced by the processing of distractors (irrelevant discs and auditory stimuli). The present study aims to assess the performance on MOT on the one hand and the modulation of ERSP by emotional prosodies on the other. Note that only the modulation of ERSP by the processing of the auditory stimuli may index the perception of emotional prosodies. Also, the stimuli for emotional prosodies were culturally adapted to the Mexican shaping of emotional expressions [32]. Thus, we expect children to have developed adequate socio-emotional knowledge of the acoustic environment. Namely, the differentiation between emotional prosodies by TD children should be primarily based on high-level cognitive processes (i.e., priors), mostly within highly variable sensory environments (i.e., human voices; please refer to Fig. 1). Thus, we expect late stages of ERSP (~ 0.4–1 s post-stimulus) to index emotion processing, which capture thorough affective evaluations after previous coarser decoding. In that sense, emotion processing beyond the detection of stimuli’s physical features should be highlighted by ERSP.

### Task-relevant performance: tracking capacity (TC)

#### Definition

The tracking capacity index (*m*) that represents the ability to track the target disc during motion was extracted from the number of correct responses, after correction for guessing. Particularly, the proportion of correct responses (*p*) was defined by Eq. (1) [53]:

1 $$p= n\left/ d\right. (m\left/n \right. +d \left/ 2 \right.)$$

where *n* is the number of targets, *d* is the number of discs, and *m* is the number of targets correctly tracked. Therefore, *m* in range [$-d\!\left/ 2\right.\; d\!\left/2\right.$] was expressed by Eq. (2):

2 $$m=n(dp-d\left/ 2\right.)$$

### Statistical analysis

A mixed within-between two-way ANOVA was conducted using R-4.3.1 to assess the effects of group (ASD versus TD), voice (human, level 1, and level 2), and of the interaction between factors on *m*: group × voice with “voice” as the repeated (within) factor and “group” as the independent (between) factor. Normality of residuals was confirmed by the Shapiro–Wilk test (*W* = 0.99, *p*-value = 0.10), and sphericity was validated by Mauchly’s test (*W*_voice_ = 0.97, *p*-value_voice_ = 0.27; *W*_interaction_ = 0.97, *p*-value_interaction_ = 0.27). The ANOVA was conducted without outliers to evaluate their influence on statistical significance. The model was robust to influential values. In case of non-significance (*p*-value > 0.05), JASP [54] was used to perform a Bayesian ANOVA so that the evidence in favor of the null hypothesis (i.e., all means are equal, considering the residual variance) (BF_01_) was quantified (uniform prior modeling).

### Emotion perception: electroencephalography

#### Recording and processing

Twenty-four active electrodes placed following the extended 10–20 system were used to record continuous EEG: Fp1, Fp2, AFz, F7, F3, Fz, F4, F8, T7, C3, Cz, C4, T8, CPz, A1, P7, P3, Pz, P4, P8, A2, POz, O1, and O2. The signal was acquired at a 500-Hz sampling rate by the mBrain Train Smarting mobi semi-dry setup. The common mode sense (reference) electrode was located at FCz, and the driven right leg electrode (ground) was located at Fpz during online recording. Impedance was kept below 10 kΩ.

Preprocessing and processing were performed using EEGLab toolbox [55] version 2021.1 from MATLAB [56]. Data were offline average re-referenced. Although the low sensor density may induce the lack of representativity of the ideal “zero reference,” average referencing would subtract globalized topographical data to outline localized activity. Channel baseline means were subtracted, and 60-Hz line noise was removed implementing the Cleanline plug-in. Low-frequency drifts were withdrawn by a forward–backward finite impulse response filter (Kaiser window, transition band [0.25 0.75] Hz stopband attenuation 80 dB, clean_rawdata function). High-variance spontaneous artifacts were corrected using the artifact subspace reconstruction algorithm [57]. Bad channels were detected whenever they presented: (1) more than 5 s flatline, (2) higher line noise data than four standard deviations of the total channel population, or (3) joint probability exceeding three standard deviations from the average of the probability density function of the total channel sample. Bad channels were removed, and the superfast spherical spline interpolation method was used to interpolate them (*m* = 4, *n* = 7). On average, 1.7 channels were considered bad in both groups. The signal was then high pass filtered using Butterworth 8th order with a cutoff frequency of 1 Hz, to further run the extended Infomax into independent components analysis. Components were transferred on non-Butterworth-filtered data, and the ones labeled within the “brain” category with less than 70% confidence by the ICLabel plug-in were rejected (constant fixed-sourced artifacts removal). On average, 2.1 (ASD) and 1.78 (TD) components were removed.

Time-locked (to auditory stimuli) epochs from 1.1 s pre- to 2 s post-stimulus were extracted from the preprocessed data. Then, ERSP were extracted between 4 and 90 Hz by Morlet wavelet expanding from 3 (at 4 Hz) to 67.5 cycles (at 90 Hz) and referenced to the [-1100 -100] ms silent baseline. Namely, a surrogate distribution was created by averaging 200 random samples from the interval between trials and was used to divide data from generated epochs. Permutation statistics were then conducted to depict significant ERSP compared to baseline (1000 iterations, *p*-value < 0.05). Non-significant data were set to 0. For each participant, ERSP were averaged over trials of the same emotion. Auditory stimuli appeared at random moments of the visual task, therefore averaging over trials faded non-time-locked activity so that ERSP mostly convey information about emotion processing rather than additional cognitive processing induced by the task. Note that we expect the emotional response to be indexed beyond temporal sensors and early stages, outlining the involvement of higher-level cognitive affective representations.

### Statistical analysis

A mixed within-between three-way ANOVA was conducted to assess the effects of group (ASD versus TD), emotion (anger, disgust, fear, happiness, neutral, sadness), and voice (human, level 1, and level 2) on ERSP. Specifically, a group × emotion × voice ANOVA with “emotion” and “voice” as the repeated (within) factors and “group” as the independent (between) factor was implemented. The effects of interactions between factors were also evaluated: (a) between group and emotion, (b) between group and voice, (c) between emotion and voice, and (d) three-way interaction between group, emotion, and voice. To assess post hoc simple two-way interactions, the factor “group” acted as the moderator. As a result, the effects of emotion, voice, and their interaction were assessed on ERSP for each group independently. Then, the significant two-way interaction was followed up by the assessment of the effect of emotion at every level of voice. Finally, significant effects were complemented by two-sided pairwise comparisons.

A non-parametric cluster-based permutation approach was conducted, using Fieldtrip Toolbox [58], so that statistical effects could be related to channel × frequency × time windows (i.e., clusters). Effects were computed within the 3-dimensional matrix at each sample, for which a test statistic was computed and compared to the critical threshold (*p*-value < 0.05). Adjacent samples with statistics exceeding the threshold were grouped within a cluster. Temporal and spectral vicinity were trivially established, and clusters were composed of 1 or more neighboring sensors. Then, a cluster-level statistic was defined as the sum of statistics within every cluster. The inferential stage was established by the Monte-Carlo estimate with 1000 random permutations under the null hypothesis of exchangeability. For every iteration, the cluster-formation stage was repeated, and the maximum cluster-level statistic was stored to build a distribution. The cluster-level statistic defined from the observed data was further located on the empirical distribution. The percentage of permutations that led to a larger statistic was the *p*-value considered to assess the effect. Therefore, because a single value (the cluster-level statistic) whose probability under the null hypothesis was considered instead of the 3-dimensional matrix, multiple comparison issues were exempted. When cluster-based permutation tests were repeated (for post hoc comparisons), *p*-values were corrected with the Bonferroni method. Finally, the significance was adjusted for two-sided tests (*p*-value < 0.025).

Bayesian models on ERSP averaged within non-significant clusters were conducted using JASP to assess the evidence towards the null hypothesis (i.e., all means are equal, considering the residual variance; BF_01_). Priors were uniformly distributed for ANOVA, and a Cauchy distribution was implemented for pairwise comparisons. The normality of residuals (ANOVA) and of differences (pairwise comparisons) were evaluated by Q-Q plots (observed quantiles as regards theoretical ones) and by Shapiro–Wilk’s test. Models were not robust to outliers as normality was not met in their presence, and outputs had different interpretations. Therefore, results from models that did not include outliers were heeded. Furthermore, sphericity was validated by Mauchly’s test (*p*-value > 0.05) for all within-subject effects.

### Sample size estimation and post hoc sensitivity power analysis

#### Tracking capacity (TC)

Sample size estimation for a mixed within-between two-way ANOVA (group × voice with “voice” as the repeated factor) was conducted in G*Power [59] according to 4 predictors: (1) Cohen’s effect size, (2) minimum correlation between paired samples set at 0.5, (3) power of 0.9, and (4) *α* error probability of 0.05. Cohen’s *f* was predicted based on a similar previous study in which the performance on a unique target 4-disc MOT was analyzed for ASD and TD children [33]. At least 21 children in each group were required to reach 90% power. A post hoc sensitivity analysis highlighted that 99% power was reached for the effect of group, and 21 participants were needed to reach 90% power. Besides, 78% power was achieved for the effect of voice (non-significant, 55 children were required to reach 90%), and 72% was reached for the interaction between factors (non-significant, 63 participants were needed to reach 90%).

### Event-related spectral perturbations (ERSP)

An a priori power analysis for cluster-based permutation mixed within-between 3-way ANOVA (group × emotion × voice ANOVA with “emotion” and “voice” as the repeated factors) modeling was conducted using Fieldtrip, based on open-source codes shared by Wang and Zhang [60]. Five predictors were considered: (1) means, (2) standard deviations, (3) minimum correlation between paired samples set at 0.5, (4) power of 0.9, and (5) *α* error probability of 0.05. The means, standard deviations, and channel × frequency × time windows for significant cluster simulation were predicted from similar previous studies in which the processing of emotional prosodies from MESD was analyzed [31, 33]. A minimum sample size of 29 participants in each group was expected to reach at least 90% power. A post hoc sensitivity analysis revealed that 99% power was reached for the 3-way interaction, and at least 16 participants were needed to reach 90% power. For the effect of voice, the minimum sample size was 57 to reach 90% power (this effect was not significant). For all other effects to reach 90% power, at least 35 children were required.

## Results

### Task-relevant performance: tracking capacity (TC)

**Fig. 3:**
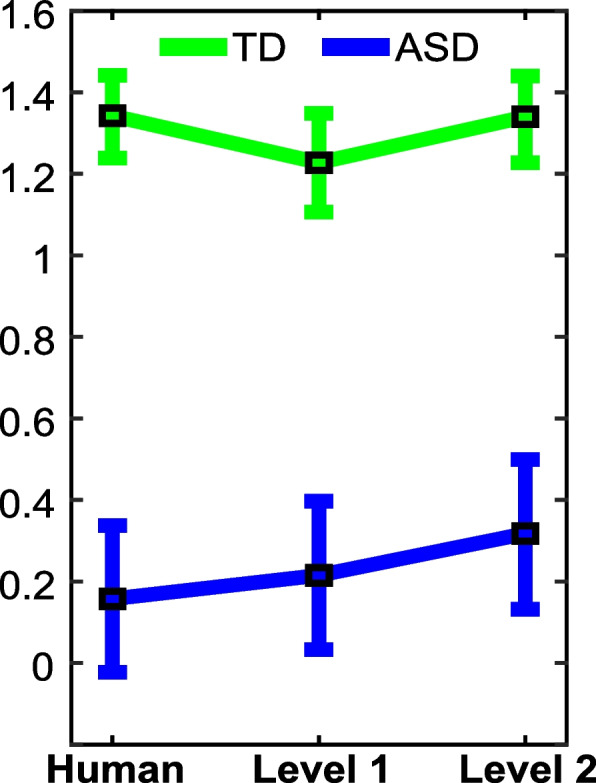
Performance on MOT (*m*) of TD and ASD children while listening to human, level 1, and level 2 voices (emotional prosodies). Error bars represent the standard errors of means (sample standard deviation divided by the square root of the number of samples)

The *m* index is provided in Fig. 3 for both groups while listening to every voice (means and standard deviations). A significant effect of group towards higher scores earned by TD children was outlined (*F*(1,78) = 29.25, *p*-value < 0.001, *η*^2^_*G*_ = 0.25). However, the effects of voice and of the interaction between group and voice did not reach significance (*F*_voice_(2,156) = 1.62, *p*-value_voice_ = 0.20, *η*^2^_*G*-voice_ = 0.002; *F*_interaction_(2,156) = 1.22, *p*-value_interaction_ = 0.3, *η*^2^_*G*-interaction_ = 0.002). The Bayesian mixed ANOVA outlined evidence towards the null hypothesis for the voice effect (BF_01_ = 5.59, moderate), but highlighted evidence towards the alternative hypothesis for the voice + group + voice × group model (BF_01_ = 0.002, very strong). Post hoc analysis that corrects for multiple testing by setting the prior probability that the null hypothesis was asserted across comparisons at 0.5 revealed evidence for the null hypothesis for all pairwise comparisons within the voice factor for both TD and ASD and evidence towards the alternative hypothesis for the ASD versus TD comparison.

### Emotion perception: electroencephalography

Significant statistical outputs for the main effects of group, emotion, and interactions are detailed in Table 2. Besides, the effect of voice was not significant; however, two clusters of channel × frequency × time windows of interest are presented. Overall, significance corresponded to midline frontal, anterior frontal, central, parietal, centroparietal, and parieto-occipital sensors, from theta to gamma waves and from 0 to 1.45 s post-stimulus. Bayes factors that indicate the likelihood of the data to occur under the null hypothesis as compared to the alternative hypothesis (BF_01_) are also provided in case of non-significant results. Coefficients above 1 highlight data in favor of the null hypothesis. Note that hereafter, the terminology “emotion differentiation” refers to the modulation of ERSP by emotional prosodies rather than the behavioral labeling of emotions.

**Table 2:** Statistical outputs for three-way main effects of group, emotion, voice, and interactions

Effect	Sum(*F*)	*p*-value	*η*^2^_*G*_	BF_01_	Time (s)	Frequency (Hz)	Topography
Group, *F*(1,78)	61,721	0.001	0.16***	N/A	[0 0.40]	[4 58]	Cz, Pz, CPz, POz
	29,247	0.018	0.15***	N/A	[0 0.44]	[4 47]	Fz, AFz
Emotion, *F*(5,390)	27,517	0.028	0.11**	N/A	[0 1.45]	[48 68]	Cz, Pz, CPz, POz
Voice, *F*(2,156)	36,186	0.063	0.01*	0.26*	[0 0.49]	[5 30]	Fz, AFz
	33,619	0.070	0.004*	12.17**	[0 0.52]	[5 45]	Cz, Pz, CPz, POz
Group × emotion, *F*(5,390)	49,265	0.009	0.21***	N/A	[0 1.45]	[4 13]	
Group × voice, *F*(2,156)	65,617	0.003	0.25***	N/A	[0 1.05]	[4 71]	
	37,698	0.009	0.19***	N/A	[0 0.32]	[4 53]	Fz, AFz
Emotion × voice, *F*(10,780)	542,015	0.001	0.32***	N/A	[0 1.45]	[4 90]	Cz, Pz, CPz, POz
	125,101	0.002	0.28***	N/A	[0.34 1.45]	[4 12]	Fz, AFz
	60,673	0.022	0.16***	N/A	[0 1.45]	[50 79]	
	59,966		0.15***	N/A		[12 20]	
Group × emotion × voice, *F*(10,780)	178,231	0.001	0.35***	N/A		[4 90]	Cz, Pz, CPz, POz
	35,239	0.004	0.26***	N/A		[14 47]	Fz, AFz

Code for Bayes factor: anecdotal, “.”; moderate, “*”; strong, “**”; very strong, “***”; extreme, “****”. BF_01_ < 0 highlights data in favor of the alternative hypothesis (bold). Code for *η*^2^_*G*_: small, “*”; medium, “**”; large, “***”. Sum(*F*) is the sum of *F*-values within the cluster (i.e., the test statistic). Degrees of freedom of the *F*-value are also indicated (factor: numerator; residuals: denominator)

Significant post hoc two-way effects are presented in Table 3. Emotion and voice effects were not significant in TD; however, non-significant clusters of interest are presented.

**Table 3:** Statistical outputs for post hoc simple two-way effects in ASD and TD

Group	Effect	Sum(*F*)	*p*-value	*η*^2^_*G*_	BF_01_	Time (s)	Frequency (Hz)	Topography
ASD	Emotion, *F*(5,195)	49,553	0.034	0.12**	N/A	[0 1.45]	[43 69]	Cz, Pz, CPz, POz
		39,731	0.045	0.07**	N/A	[0 1.28]	[4 11]	
	Voice, *F*(2,78)	94,962	0.030	0.15***	N/A	[0 0.73]	[4 78]	
		78,351	0.040	0.07**	N/A	[0 0.54]	[4 53]	Fz, AFz
	Emotion × v oice, *F*(10,390)	265,230	0.002	0.26***	N/A	[0 1.45]	[18 90]	Cz, Pz, CPz, POz
		143,084	0.005	0.18***	N/A		[4 24]	Fz, AFz
		83,700	0.016	0.14***	N/A	[0.49 1.45]	[4 13]	Cz, Pz, CPz, POz
TD	Emotion, *F*(5,195)	2323	0.972	0.08**	0.005****	[0.58 1.25]	[4 7]	
		1768	0.996	0.02*	0.11*	[0.05 0.21]	[5 7]	Fz, AFz
	Voice, *F*(2,78)	10,638	0.420	0.04*	0.04**	[0.93 1.43]	[6 9]	
		7257	0.679	0.01*	2.92	[0.16 0.62]	[4 6]	Cz, Pz, CPz, POz
		6745	0.731	0.03*	0.007****	[1.10 1.45]	[21 30]	Fz, AFz
		2370	0.998	0.09**	< 0.001****	[0.07 0.35]	[7 9]	
	Emotion × v oice, *F*(10,390)	161,965	0.002	0.12**	N/A	[0 1.45]	[4 22]	Cz, Pz, CPz, POz
		150,752		0.14***	N/A		[21 90]	Fz, AFz
		140,380		0.17***	N/A		[21 59]	Cz, Pz, CPz, POz
		49,475	0.03	0.13***	N/A	[0.24 1.21]	[4 9]	Fz, AFz

Code for Bayes factor: anecdotal, “.”; moderate, “*”; strong, “**”; very strong, “***”; extreme, “****”. BF_01_ < 0 highlights data in favor of the alternative hypothesis (bold). Code for *η*^2^_*G*_: small, “*”; medium, “**”; large, “***”. Sum(*F*) is the sum of *F*-values within the cluster (i.e., the test statistic). Degrees of freedom of the *F*-value are also indicated (factor: numerator; residuals: denominator)

The significant two-way interaction was followed up by the assessment of the effect of emotion at every level of voice. Significant statistical outputs are presented in Table 4. The emotion effect was not significant for human and level 1 voices in ASD nor in TD. It was not significant for level 2 voice in TD either. Nonetheless, non-significant clusters of interest are presented.

**Table 4:** Statistical outputs for post hoc simple main effect of emotion in ASD and TD at every level of voice

Group	Voice	Sum(*F*)	*p*-value	*η*^2^_*G*_	BF_01_	Time (s)	Frequency (Hz)	Topography
ASD	Human, *F*(5,195)	7057	0.390	0.03*	14.18**	[0.95 1.45]	[10 15]	Cz, Pz, CPz, POz
		2832	0.889	0.10**	0.25	[0.25 0.78]	[17 24]	
		2232	0.952	0.11**	1.72	[0 0.23]	[31 37]	
		1794	0.990	0.06**	0.50	[0.40 0.79]	[19 22]	Fz, AFz
		1532	0.994	0.06**	0.71	[0.55 0.77]	[16 23]	Pz, POz
		1513	0.994	0.07*	5.37*	[0.42 0.66]	[8 10]	
	Level 1, *F*(5,195)	19,324	0.128	0.03*	15.53**	[0.05 1.45]	[42 69]	Cz, Pz, CPz, POz
		7864	0.711	0.05*	5.46*	[0.22 0.65]	[4 7]	
		3653	1	0.06**	2.34	[0.76 1.28]	[5 11]	Cz, Pz, CPz
		2148	1	0.05*	4.41*	[0.22 0.46]	[10 13]	Fz, AFz
		1844	1	0.06**	3.10*	[1.03 1.45]	[80 85]	Cz, Pz, CPz, POz
		1704	1	0.11**	0.16*	[0.02 0.2]	[6 9]	Pz, CPz, POz
		1471	1	0.05*	3.51*	[0 0.42]	[20 25]	
	Level 2, *F*(5,195)	66,904	0.033	0.08**	N/A	[0 1.45]	[43 77]	Cz, Pz, CPz, POz
		31,350	0.048	0.08**	N/A	[0 1.16]	[16 32]	
TD	Human, *F*(5,195)	3672	0.762	0.03*	14.20**	[0.10 0.49]	[7 12]	Cz, Pz, CPz
		2578	0.935	0.05*	6.23*	[0.77 1.04]	[4 5]	Pz, POz
		2155	0.973	0.04*	0.28*	[0.47 0.90]	[8 11]	Cz, Pz, CPz
		2044	0.979	0.11**	0.10**	[0 0.31]	[7 9]	Fz, AFz
		1555	0.997	0.09**	0.25*	[0.6 1.23]	[38 42]	Cz, Pz, CPz, POz
		1500	0.998	0.05*	6.88*	[0.10 0.45]	[24 27]	Fz, AFz
		1379	0.998	0.05*	2.70	[0.28 0.67]	[22 27]	Cz, Pz, CPz, POz
	Level 1, *F*(5,195)	3853	0.743	0.07**	0.05**	[0.14 0.51]	[4 5]	Fz, AFz
		2208	0.976	0.05*	0.27*	[0 0.19]	[4 5]	Cz, Pz, CPz
		1672	0.995	0.11**	2.66	[1.1 1.3]	[7 10]	
	Level 2, *F*(5,195)	8273	0.210	0.09**	0.31*	[0.02 0.52]	[9 14]	Fz, AFz
		5161	0.538	0.12**	0.04**	[0.82 1.22]	[16 21]	Cz, Pz, CPz, POz
		3498	0.820	0.08**	2.85	[0.66 1.03]	[4 5]	Fz, AFz
		2848	0.916	0.10**	0.06**	[0.18 0.77]	[20 23]	Cz, Pz, CPz, POz
		2556	0.953	0.07**	0.18*	[0.04 0.34]	[9 14]	Cz, Pz, CPz

Code for Bayes factor: anecdotal, “.”; moderate, “*”; strong, “**”; very strong, “***”; extreme, “****”. BF_01_ < 0 highlights data in favor of the alternative hypothesis (bold). Code for *η*^2^_*G*_: small, “*”; medium, “**”; large, “***”. Sum(*F*) is the sum of *F*-values within the cluster (i.e., the test statistic). Degrees of freedom of the *F*-value are also indicated (factor: numerator; residuals: denominator)

The significant emotion effect for level 2 in ASD was followed up by the assessment of pairwise comparisons between emotions. Significant statistical outputs are presented in Table 5. Non-significant clusters of interest are also provided.

**Table 5:** Statistical outputs for post hoc simple pairwise comparisons for level 2 in ASD

Comparison	Sum(*T*)	*p*-value	*d*	BF_01_	Time (s)	Frequency (Hz)	Topography
Anger-disgust	2499	0.464	0.46*	0.19*	[0.05 1.18]	[45 53]	Cz, Pz, CPz, POz
	5410	0.26	0.28*	1.46	[0.47 1.45]	[21 28]	
	1636	0.59	0.08*	4.74*	[0.64 1.05]	[30 34]	
Anger-fear	31,858	0.024	0.56***	N/A	[0 1.45]	[15 42]	
Anger-happiness	− 7892	0.207	− 0.46*	0.42		[55 68]	
	− 630	0.862	− 0.24*	0.03***	[0.80 1.04]	[51 54]	
	16,112	0.097	0.23*	2.19	[0.01 1.12]	[10 26]	
	1046	0.73	0.02*	4.80*	[0 0.32]	[32 37]	
	636	0.83	0.35	1.21	[1.31 1.45]	[31 36]	
Anger-neutral	− 30,062	0.032	− 0.28*	2.03	[0 1.45]	[50 73]	
	− 1494	0.746	− 0.40*	**0.63**	[0 0.38]	[70 79]	
	2858	0.51	0.27*	3.09*	[0.25 0.75]	[27 37]	
	− 2514	0.57	− 0.27*	1.65	[0.84 1.33]	[38 45]	
	− 15,912	0.06	− 0.50**	0.21*	[0.64 1.42]	[4 7]	
	− 15,341	0.06	− 0.50**	0.24*	[0 0.38]	[4 13]	
Anger-sadness	− 4922	0.29	− 0.27*	1.59	[0 1.45]	[56 63]	
	− 6764	0.22	− 0.20*	2.95	[0.30 1.37]	[33 45]	
	− 12,466	0.10	− 0.17*	3.41	[0.64 1.34]	[4 11]	
	1430	0.74	0.32*	1.24	[0.01 0.34]	[26 32]	
Disgust-fear	17,662	0.054	0.032*	5.28*	[0 1.31]	[15 32]	
	748	0.940	0.37*	**0.58**	[0.01 0.38]	[67 75]	
	4262	0.38	0.35*	0.62	[0.15 0.50]	[4 7]	
Disgust-happiness	12,027	0.103	0.29*	1.66	[0 1.29]	[13 26]	
	− 4270	0.410	− 0.28*	1.42	[0.50 1.45]	[46 54]	
	− 1968	0.76	− 0.70**	0.02***	[0.41 1.41]	[63 70]	
Disgust-neutral	− 64,638	0.009	− 0.66***	N/A	[0 1.45]	[35 74]	
	− 12,592	0.11	− 0.78**	0.004****	[0.47 1.14]	[4 8]	
Disgust-sadness	− 18,609	0.064	− 0.58 **	0.04**	[0.35 1.45]	[22 44]	
Fear-happiness	30,744	0.014	0.62**	N/A	[0.06 1.45]	[5 20]	
	− 26,413	0.017	− 0.58**	N/A	[0 1.45]	[40 73]	
Fear-neutral	− 87,025	0.002	− 0.54**	N/A		[37 90]	
	− 48,078	0.01	− 0.61**	N/A		[4 9]	
	− 41,269		− 0.67**	N/A		[15 35]	
Fear-sadness	− 41,286	0.015	− 0.48*	N/A		[20 55]	
Happiness-neutral	− 68,891	0.004	− 0.61**	N/A		[4 13]	
	− 22,530	0.04	− 0.48*	0.42	[0 1.23]	[15 30]	
	− 7802	0.19	− 0.07*	4.64*	[0 1.45]	[54 65]	
Happiness-sadness	− 36,292	0.017	− 0.56**	N/A	[0.19 1.31]	[5 21]	
	− 17,532	0.06	− 0.28*	1.81	[0.46 1.45]	[30 45]	
	2167	0.58	0.54**	0.09*	[0.90 1.45]	[60 70]	
	− 30,031	0.022	− 0.52**	N/A	[0.41 1.42]	[5 22]	Fz, AFz
	− 4188	0.35	− 0.20*	2.89	[0.39 1.43]	[34 39]	
Neutral-sadness	36,553	0.009	0.51***	N/A	[0 1.45]	[42 80]	Cz, Pz, CPz, POz
	− 8168	0.183	− 0.20*	2.93	[0.26 1.45]	[31 41]	

Code for Bayes factor: anecdotal, “.”; moderate, “*”; strong, “**”; very strong, “***”; extreme, “****”. BF_01_ < 0 highlights data in favor of the alternative hypothesis (bold). Code for Cohen’s *d*: small, “*”; medium, “**”; large, “***”. Sum(*T*) is the sum of *T*-values within the cluster (i.e., the test statistic)

**Fig. 4:**
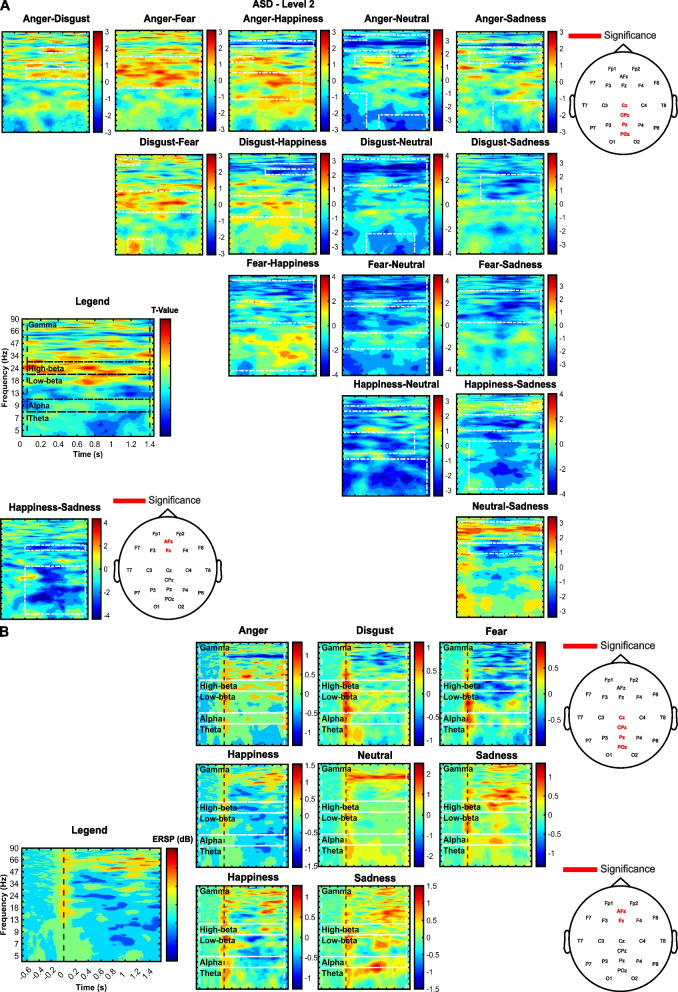
Processing of level 2 voices by ASD. **A** T-values for pairwise comparisons through time and frequencies, averaged over channels of significance (indicated by the legend “Significance”). The “happiness-sadness” comparison appears twice: the first (4th row from the top, last column from the left) provides values averaged over Cz, Pz, CPz, and Poz, and values were averaged over Fz and AFz, within the second plot (5th row from the top, 1st column). Clusters mentioned in Table 5 are outlined by white dotted rectangles. **B** ERSP (dB) are presented through time and frequencies, averaged over Cz, Pz, CPz, POz and Fz, and AFz where relevant (happiness and sadness)

A graphical representation of test statistics for pairwise comparisons (*t*-value) and clusters provided in Table 5 are outlined (Fig. 4A). Also, ERSP while listening to emotional prosodies uttered by level 2 voices are provided (Fig. 4B).

**Fig. 5:**
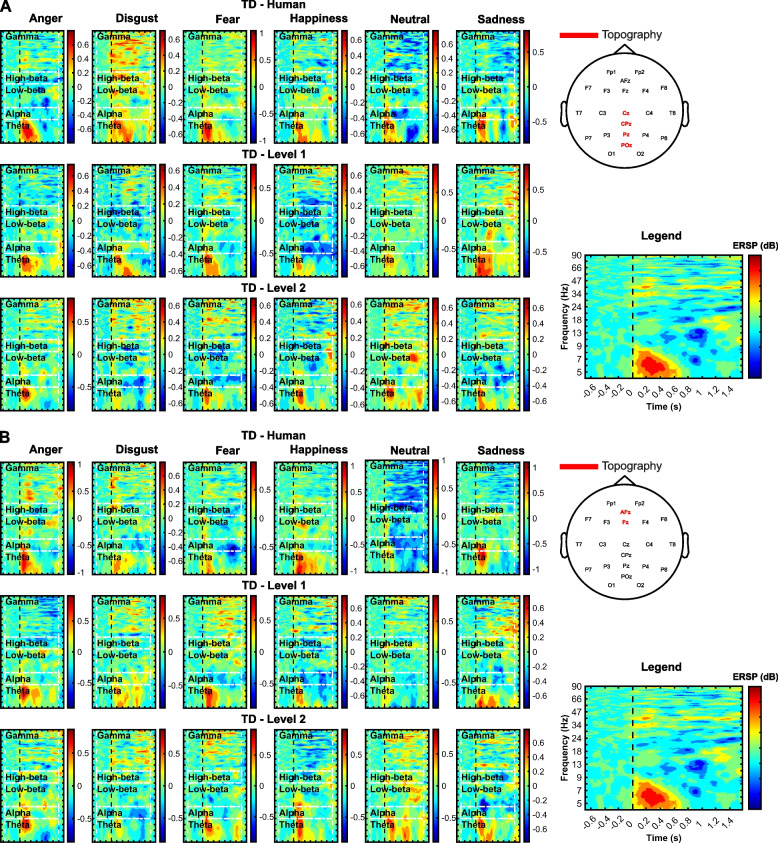
Processing of human, level 1, and level 2 voices by TD. **A** ERSP (dB) are presented through time and frequencies, averaged over Cz, Pz, CPz, and POz. **B** ERSP (dB) are presented through time and frequencies, averaged over Fz and AFz

Pairwise comparisons after non-significant frequentist analysis where the alternative hypothesis was favored by Bayes factors are available in Additional file 1. Particularly, statistical outputs for level 1 in ASD are provided in Additional file 1: Table S1. Bayesian statistics outlined evidence in favor of the alternative hypothesis for anger-sadness (moderate), anger-happiness, disgust-happiness, and happiness-sadness (anecdotal). Statistical outputs for TD are presented in Additional file 1: Table S2 (human), Table S3 (level 1), and Table S4 (level 2). Bayesian statistics highlighted evidence in favor of the differentiation of unique patterns of emotions at every level. Figure 5 presents the ERSP while processing emotions uttered by human, level 1, and level 2 voices (TD), and Table 6 specifies the pairwise comparisons for which the alternative hypothesis was favored (✓) or disfavored (X) in TD.

**Table 6:** Pairwise comparisons for which the alternative hypothesis of emotion differentiation was favored (✓) or disfavored (X) in TD

Comparison	Human	Level 1	Level 2
Anger-disgust	✓	✓	✓
Anger-fear	✓	✓	✓
Anger-happiness	✓	✓	X
Anger-neutral	✓	✓	✓
Anger-sadness	✓	✓	✓
Disgust-fear	X	X	X
Disgust-happiness	✓	✓	X
Disgust-neutral	✓	✓	✓
Disgust-sadness	✓	X	X
Fear-happiness	✓	X	✓
Fear-neutral	✓	✓	✓
Fear-sadness	✓	X	✓
Happiness-neutral	X	✓	✓
Happiness-sadness	X	✓	✓
Neutral-sadness	X	✓	✓

### Sensitivity analysis: age- and gender-controlled sample

A sensitivity analysis was conducted to control age and gender, which differed between ASD and TD and may have biased statistical outputs [61]. Particularly, statistical analyses on TC and ERSP were performed again with an age- and gender-controlled sample selected from the original data. Details of these analyses are provided in Additional file 1 within the “Sensitivity analysis: age- and gender-controlled sample” section, Table S5, and Table S6. The results previously presented were robust to age and gender differences.

## Discussion

This study aimed to assess the differentiation of emotional prosodies during implicit perception under human and naturalness-reduced voices (i.e., decreased variability of acoustic components). Perceptual abilities were further underpinned by the behavioral TC performance (task-relevant). Primarily, the increase and decrease of power across time and frequencies of EEG data induced by the processing of a stimulus, which corresponds respectively to the synchronization and desynchronization of neural firing (ERSP), was analyzed to portray rhythmic cortical oscillations associated with emotion processing. Fundamentally, ERSP time-locked to the apparition of auditory information (rather than to visual stimuli) captured the perception of the emotion prosody and canceled out the grip on auxiliary cognitive processes. Of important note, selective attention processes were required from MOT for which performance by autistic children has been previously outlined to be unbiased from dynamic motion hindrance [62]. Therefore, the TC allowed to infer about sensory sampling relative to prior knowledge on the informativeness of target and distractor dots. However, inferences about precision-weighting mechanisms may not be directly extended to the processing of the auditory stimuli. Rather, they were engineered to create more reliable acoustic environments to promote emotion differentiation by autistics.

Consistent with our hypothesis, (a) autistic children presented poorer performance than TD on MOT, displaying a behavioral marker of excessive weight ascribed to the sensory processing of noisy information. Second, (b) improved emotion differentiation by ASD was highlighted while naturalness in voice was reduced. This finding outlines the ability of ASD to discriminate acoustic patterns for emotion differentiation in speech when stimuli are simpler. Nevertheless, (c) ERSP were partially congruent with foreseen patterns, revealing different neural processes compared to typically expected. Finally, (d) the neurophysiological data aligned with the successful emotion differentiation by TD children, highlighted by Bayesian statistics (rather than significant frequentist *p*-value). This study provides neurobiological insights into the facilitation of emotion differentiation by acoustically modified speech in autistic children.

First, answering MOT requires selective attention processes that implies adjusting sensory processing to the most salient information, in this case, optimizing the precision to affordances from distractors to focus on the targeted dot. In line with the Bayesian modeling of selective attention, moving dots are predicted visual inputs (priors) whose trajectories, speed, and number should not trigger a significant shift (update) in prior knowledge because of no unexpected information gain across trials [14]. Therefore, the precision to prediction errors should be low, and perception should be narrowed towards prior probabilistic learnings of stimuli apparition. Nonetheless, a substantial factor is the degree of cognitive demand induced by the relevant task. In a similar sample of TD and ASD children, unique-target MOT four-disc partially depleted attentional control resources of TD, dovetailing with the processing of irrelevant distractors. In this scenario, the precision to irrelevant dots was only partially weakened (unoptimized performance, but higher than on an eight-disc task of higher cognitive load), and the discrimination of irrelevant auditory emotional prosodies was increased [33]. Accordingly, the present results outline unoptimized performances of TD children. Besides, lower TC by ASD than TD were observed, suggesting the over-precision ascribed to the sensory processing of irrelevant dots or to prediction errors, leading to inefficient updating of contextual statistics towards a high learning rate of environmental volatility that impaired the performance of autistics [17]. Furthermore, strong evidence towards the concomitant effect of voice and group on TC was observed, highlighting different tendencies between ASD and TD as regards TC across voice environments. Although non-significant, a slight tendency towards better TC while reducing voice naturalness was observed in ASD. The simplified acoustic environment induced by naturalness reduction may have fostered probabilistic learnings of the sensory environment towards inferences closer to priors.

Autistic children differentiated unique patterns of emotions only when naturalness in speech was reduced, particularly while listening to level 2. Besides, a general effect of group (ASD vs TD) was also outlined, observing a neurophysiological basis of emotion processing divergent from typical perception. Also, group and voice effects acted concomitantly on ERSP patterns, an interaction emphasizing a singular effect of naturalness reduction on autistic perception. Naturalness-reduced voices have been designed to create more reliable (near to discrete) acoustic variations within words while conserving unique profiles of emotions [31]. Particularly, pitch, harmonics, formants, and lexical stress patterns followed simpler hierarchical structures than the original speech. Therefore, the perception of variations that define emotional prosodies depended to a much greater extent on the accuracy of acoustic hierarchical scales (fine-grained) recognition rather than on prior knowledge about acoustic expressions of affective states. This type of processing is nearer to previously observed in ASD where intact intonational phrase boundary recognition in structured nonspeech (such as music), but impaired in speech was outlined [21]. Indeed, similar to naturalness-reduced voices, music (contrary to speech) processing requires the accurate encoding of acoustic variations [63]. Similarly, in a study applied to tonal language, autistic children compared to TD showed enhanced recognition of fined-grained acoustic variations (within lexical tone categories) that listeners must usually learn to ignore for efficient speech comprehension [64]. Finally, by overfitting priors to precise and “noisy” information, autistics would gather knowledge optimized to decipher the emotional identities provided by naturalness-reduced voices [65].

ASD has been characterized with enhanced sensory decoding such as pitch processing of nonspeech stimuli, an advantage that reaches its peak during childhood [66, 67]. Nevertheless, high heterogeneity between studies highlighted stimulus complexity as a non-negligible factor. Indeed, performance may be worsened by decreasing the acoustic stability (e.g., isolated tones that rely upon absolute pitch had higher explanatory strength of enhanced ability than pitch interval detection underpinned by relative pitch) [66]. Acoustic intricacy may shade the heightened sensory ability to detect prosodic modulations for which only a coarse decoding of sensory evidence may be beneficial (i.e., sample of the most relevant sensory cues) [21, 67]. Indeed, greater sensitivity to acoustic variability of speech in which ambiguous variations may exist could dominate the grip on valuable cues [68]. Overall, the recognition of affective prosody by autistics may differ from matched TD, but high heterogeneity between studies was outlined as regards effect sizes and differences were no longer significant after correcting for publication bias [48]. An interesting moderator to study may be the nature of stimuli for which lower acoustic complexity in speech may benefit autistics for processing emotional prosodies. Accordingly, in the present study, more reliable acoustic environments have fostered the differentiation of emotional prosodies by autistics. Besides, the extended topography and late latency of the effect may suggest the involvement of higher-level cognitive appraisal.

Emotion differentiation was observed over midline central, parietal, centroparietal, parieto-occipital, frontal, and antero-frontal electrodes in both ASD and TD. Indeed, early emotional salience identification and representations within linguistic memory for lexical access (~ 200 ms post-stimulus), as well as later thorough affective evaluations (~ 400–1000 ms post-stimulus) have usually been observed over those cortical regions [5, 69]. The emotional response implies bilateral cortex regions embedded within functional networks including active inter-regional connections. Although hemispheric lateralization hypotheses have found empirical bedrocks (e.g., right-hemispheric dominance, valence, and motivational lateralization), the overall evidence fails to support any of the proposed frameworks, rather towards highlighting the relevance of inter-hemispheric functional hubs [70]. In that context, EEG-informed functional magnetic resonance (fMRI) imaging evidenced midline-electrode activity in response to emotion processing as a marker of activity within inter-regional and inter-hemispheric connections, including posterior parietal, ventrolateral and dorsolateral prefrontal cortices, superior frontal gyrus, premotor, anterior cingulate, and insular cortices [71]. Note however that in the present study, no explicit relationship between the engagement within an emotion processing task and ERSP patterns of emotion differentiation was assessed. Therefore, no specific processing of the emotional information may be directly inferred from the present results.

The most efficient emotion identification by autistics was observed while listening to the highest level of naturalness-reduced voices. Especially, early alpha (~ 8–12 Hz) synchronization (0–0.2 s) was observed for all emotions, except for happiness (no difference from baseline), outlining higher event-related synchronization (ERS) during anger and sadness than during happiness, and during neutral than anger. The overall cortical inhibition (alpha ERS) at early stages may outline the disengagement of irrelevant processes to further attentional and cognitive processing towards salient emotional information [72], a common observation between ASD and TD. Nevertheless, the cortical inhibition for emotion differentiation was differently modulated by the acoustic profile of prosodies (significant emotion × voice interaction) between ASD and TD. Particularly, autism may be characterized by higher inhibition under low valence (anger and sadness), whereas in TD, arousal also mediated alpha ERS (e.g., highest ERS at higher arousal such as anger and happiness). In neurotypicals, differential spectral activity in alpha was previously highlighted among emotions, allowing recognition, principally based on the arousal content [73, 74]. In that sense, alpha spectral activity encodes neural patterns of affective attention based on relevance [75]. Nevertheless, the affective salience may vary (1) within-emotion when the precision to prior or prediction error is challenged for perception, highlighted here by uneven emotion differentiation in TD as naturalness decreased, and (2) between ASD and TD when prior knowledge had mostly been fitted towards high-precise auditory information or abstract social representations, respectively. Finally, alpha event-related desynchronization (ERD) was mostly observed both in ASD and TD at later stages (0.2–1.4 s), highlighting the overall disinhibition of relevant brain regions for emotion integration once irrelevant processing had been previously disengaged.

Emotion differentiation by ASD (level 2) was outlined in theta (~ 4–7 Hz) by early (0–0.2 s) ERS (except for happiness: no difference from baseline at Cz, Pz, CPz, POz), whose power differed between emotions. Although ERS was sustained for sadness and neutral prosodies, desynchronization was observed at late stages (0.2–1.4s) for higher-arousal prosodies (anger, disgust, fear, and happiness). Similar patterns were observed in TD (ERS followed by ERD while listening to all levels of naturalness reduction), nevertheless highlighting longer ERS than ASD. Indeed, ERS was observed within [0 1 s] while listening to human and level 1 voices and within [0 0.8 s] for level 2. Theta activity observed at frontal, central, and parietal midline electrodes is involved in memory encoding, retrieval, semantic representations, and cognitive engagement observed while processing affective and linguistic information [76, 77]. The synchronization outlined here, induced by emotional speech prosody may be an index of successful appraisal for internal affective representations. Importantly, longer synchronization in TD may highlight cognitive engagement, from low-level acoustic feature processing to coarse valence/arousal/salience detection, and later complex affective state portrayal. Nevertheless, shorter ERS at level 2 (TD and ASD) may outline fewer internal representations triggered by the acoustic properties of stimuli that orient the perception towards higher precision to acoustic features encoding. Finally, late ERD may highlight less sensitivity to processing semantic representations and cognitive control before returning to baseline [76].

Low-beta rhythm (~ 13–20 Hz) observed at frontal, central, and parietal cortices may index various cognitive processes such as empathic representations, memory encoding/retrieval, and salience detection after emotional stimuli processing. Here, ASD children presented both ERS and ERD for all emotions. ERS was mostly observed at lower valence (anger, disgust, fear, and sadness) and ERD during happiness, although desynchronization also appeared at late stages for low-valence prosodies. Interestingly, similar patterns were observed for TD while listening to all levels of naturalness reduction. Nevertheless, low-beta activity did not foster emotion discrimination for TD during human and level 1 processing. Rather, the prosodic differentiation at level 2 was fostered by stable spectral and temporal acoustic patterns. First, low-beta ERD, together with theta ERS may underpin memory encoding and retrieval induced by motivational relevance of salient emotional stimuli [78]. Second, understanding others’ emotions and intentions induced by the activation of the mirror neuron network may have triggered low-beta ERD, a pattern observed during emotional stimuli perception [73, 74, 79]. Finally, concurrent low-beta ERS may have been induced by attentional allocation towards salient emotional information to ensure the access to cognitive resources needed for emotion discrimination [80].

High-beta oscillations (~ 21–30 Hz) after emotion induction are involved in the maintenance of an affective state for further internal representation elaboration, where ERS is sensitive to valence and arousal, allowing emotion identification [81]. For instance, higher high-beta power may be induced by high-arousal and low-valence stimuli [81, 82]. On the other hand, high-beta ERD may be observed after lexico-semantic access, especially when deeper semantic representations are formed [83]. Here, both high-beta ERS and ERD were observed after emotion induction. Accordingly, anger and sadness entailed both early and late ERS in ASD. Interestingly, processing disgust and happiness induced ERS at early stages, but the access to lexico-semantic information dominated emotion differentiation at later stages, and during the whole post-stimulus period when perceiving fear. Similarly, high-beta oscillations allowed emotion differentiation by neurotypicals only at level 2, mostly relying on lexico-semantic access.

Finally, gamma (> 30 Hz) ERS indicates access to executive control resources and associative memory. Higher ERS denoted lower ability to categorize and differentiate emotions emphasizing increased cognitive effort for information integration [84]. Besides, cognitive resources may also be higher while processing negative versus positive stimuli by neurotypicals [81, 85]. On the other hand, gamma ERD together with theta ERS may correlate with successful verbal memory encoding/retrieval [86], and with semantic, syntactic, and phonological processes [87]. Here, autistic children presented effortful processing (ERS), particularly for sadness, neutral, happiness, and anger, while lower demand (ERD) was highlighted for disgust and fear. As expected in TD, low-valence and highest arousal stimuli induced the highest cognitive resources (ERS) whereas ERD underlined the perception of happiness, neutral, and sadness.

Emotion differentiation by TD and ASD involved late-stage neural processing, suggesting thorough appraisal of stimuli beyond early sensory decoding. Indeed, after basic sensory processing up to around 100 ms after stimulus’ onset [88], saliency detection and auditory object categorization for emotional appraisal mostly based on valence and arousal occurs around 200 ms [89]. Finally, contextual, memory relevance, and refined representations are encoded beyond 400 ms [90]. Thus, although the naturalness reduction was solely substantiated by acoustic modulations of emotion prosodies, the emotion differentiation by autistic children may have been underpinned not only by the sensory decoding of acoustic cues but may have also triggered higher-order appraisals. Future works may be recommended to explore the specificities of sensory and cognitive engagement for prosodic differentiation. Of note, spatially informed EEG (i.e., coupled with fMRI or functional near-infrared spectroscopy) could help outline brain regions involved in low- and high-level processing. Also, correlations between ERSP, sensory and socio-emotional abilities, or explicit emotion evaluation performance may be assessed.

### Limitations

The indirect perception of emotions may raise the need for assessing the implementation of naturalness-reduced voices into direct emotion labeling tasks. One option would be to embed them into an intervention designed to improve the ability to differentiate one emotion from another and associate it with daily life contexts or other stimuli (e.g., faces, music, sounds). Naturalness could be progressively increased according to the patient’s learning towards the generalization of socio-emotional abilities to human voices. Also, although cognitive emotional appraisal was inferred from extended topography and late stages of neural data, the specificity of the association with emotional knowledge was out of the scope of this study. However, it may be interesting for future works to directly explore the link between emotion differentiation and high-order processes. For instance, the assessment of the association with specific memories or of the correlation with daily socio-emotional abilities.

Neural patterns may be interpreted with caution. Although statistical analyses promoted inferences about activity time-locked to emotional stimuli, ERSP may be partially biased by cognitive processes induced by the experimental paradigm. Nonetheless, cognitive processes external to emotion processing were constant between emotional prosodies so that statistical analyses for emotion differentiation were not biased. For instance, alpha, theta, beta, and gamma oscillations may have been influenced by executive control resources engaged in attentional reallocation processes between visual and auditory stimuli. Also, the performance on MOT may have been biased by the cognitive load induced by the processing of the auditory stimuli. Finally, the addition of a matched acoustic condition devoid of social and emotional associations would have enriched the suggestion of high-level processing related to the perception of emotional prosodies.

Some uncontrolled covariates may have biased the ability to differentiate emotional prosodies. Particularly, musical training may enhance emotion recognition in speech, underlined by shared acoustic [91] and neural [92] coding [93]. Indeed, musical therapy may be beneficial for autistics [94]. Besides, practice for recognizing low-level acoustic features in music may promote emotion differentiation induced by most naturalness-reduced voices [95]. Other variables such as parenting style of education [96], physical activity [97], interoceptive awareness [98], or intelligence quotient [61] may have shaped the responsiveness to emotional prosodies. We recommend future studies to control those possible covariates.

Finally, ERSP observed in TD did not foster significant emotion comparisons by the frequentist ANOVA, but evidence that favors the alternative hypothesis of emotion differentiation was outlined while listening to all voices (human, level 1, and level 2). Indeed, a relevant factor of disagreement between frequentist and Bayesian approaches is the effect size. Different sensitivities to this parameter usually lead to significant *p*-values for medium to large effect sizes (and small to moderate sample sizes), outlining non-significance for small effect sizes. Nevertheless, the Bayes factor may in case of small to medium effect sizes, highlight evidence that favors the alternative hypothesis, while improving the type I error (false positives) at the cost of slightly increased type II errors (false negatives) [99]. One possible explanation of the non-significance observed here (*p*-value > 0.05) may be the semi-randomized order for voice presentation (13 children in each group started the paradigm by listening to a particular voice). In the case of TD, the repetition of the MOT task may have encouraged the reduction of cognitive load, leading to more efficient suppression of processing task-irrelevant stimuli (i.e., the emotional prosodies). To test this hypothesis, a three-way ANOVA (emotion × voice × order) was considered. A priori power analysis for emotion effect in each group (factor order) revealed that at least 24 children were expected to reach 90% power. Further post hoc analysis revealed that 49% power was achieved with 13 children, and 30 children were required to reach 90% power. Therefore, the low sample size impeded to ascertain the effect of the order on ERSP; however, future works should consider this variable when designing similar experimental paradigms.

## Conclusions

Autistic children showed improved differentiation of emotional prosodies’ patterns in speech when the variability and volatility of temporal and spectral cues was reduced. On the other hand, the behavioral data emphasized the over-precision ascribed to sensory processing or prediction error relative to prior knowledge. Although emotions were differentiated within more stable acoustic environments, ERSP extracted from EEG data may suggest (1) atypical patterns of cortical inhibition (highest under low valence), (2) fewer memory and internal representation encoding outlined by shorter theta ERS, (3) salience detection outlined by low-beta ERS, (4) maintenance of affective state emphasized by high-beta ERS, and (5) effortful cognitive processing highlighted by gamma ERS. This study outlined the potential of reliable acoustic environments to foster emotion differentiation by autistics. Future works may assess their application within interventions to stimulate the learning of associations between acoustic patterns and higher-order socio-emotional knowledge.

### Supplementary Information

**Additional file 1. **An additional file is available that contains (1) statistical results from pairwise comparisons after non-significant frequentist analysis where the alternative hypothesis was favored by bayes factors (2) statistical results from the sensitivity analysis to control age and gender differences between ASD and TD. A PDF file is provided that includes the following tables. Table S1. Statistical outputs for post-hoc simple simple pairwise comparisons for level 1 in ASD. Code for bayes factor: Anecdotal: “.”, moderate: “*”, strong: “**”, very strong: “***”, extreme: “****”. BF01 < 0 highlights data in favor of the alternative hypothesis (bold). Code for Cohen’s d: small: “*”, medium: “**”, large: “***”. Sum(T) is the sum of T-values within the cluster (i.e., the test-statistic). Table S2. Statistical outputs for post-hoc simple simple pairwise comparisons for human voice in TD. Code for bayes factor: Anecdotal: “.”, moderate: “*”, strong: “**”, very strong: “***”, extreme: “****”. BF01 < 0 highlights data in favor of the alternative hypothesis (bold). Code for Cohen’s d: small: “*”, medium: “**”, large: “***”. Sum(T) is the sum of T-values within the cluster (i.e., the test-statistic). Table S3. Statistical outputs for post-hoc simple simple pairwise comparisons for level 1 in TD. Code for bayes factor: Anecdotal: “.”, moderate: “*”, strong: “**”, very strong: “***”, extreme: “****”. BF01 < 0 highlights data in favor of the alternative hypothesis (bold). Code for Cohen’s d: small: “*”, medium: “**”, large: “***”. Sum(T) is the sum of T-values within the cluster (i.e., the test-statistic). Table S4. Statistical outputs for post-hoc simple simple pairwise comparisons for level 2 in TD. Code for bayes factor: Anecdotal: “.”, moderate: “*”, strong: “**”, very strong: “***”, extreme: “****”. BF01 < 0 highlights data in favor of the alternative hypothesis (bold). Code for Cohen’s d: small: “*”, medium: “**”, large: “***”. Sum(T) is the sum of T-values within the cluster (i.e., the test-statistic). Table S5. Balance assessment before and after matching considering the “age” variable on the boy-only subsample. Table S6. Statistical outputs for main effects of group, emotion, voice, and interactions on the age- and gender-controlled sample. Code for bayes factor: Anecdotal: “.”, moderate: “*”, strong: “**”, very strong: “***”, extreme: “****”. BF01 < 0 highlights data in favor of the alternative hypothesis (bold). Degrees of freedom of the F value are also indicated (factor: numerator, residuals: denominator).

## Data Availability

Human and naturalness-reduced voices that support the findings of this study are openly available in Mendeley Data at http://dx.doi.org/10.17632/cy34mh68j9.5 under the following DOI: 10.17632/cy34mh68j9.5 [100].

## Abbreviations

ADOS-2: Autism Diagnostic Observation Schedule, Second Edition
AL: Atypical language
AS: Adult socialization
ASD: Autism spectrum disorder
ASRS: Autism Spectrum Rating Scales
AT: Attention
BR: Behavioral rigidity
DSM-5: Diagnostic and Statistical Manual of Mental Disorders, Fifth Edition
EEG: Electroencephalography
ERD: Event-related desynchronization
ERS: Event-related synchronization
ERSP: Event-related spectral perturbations
MESD: Mexican Emotional Speech Database
MOT: Multiple object tracking
PS: Peer socialization
SC: Social/communication
SER: Social/emotional reciprocity
SR: Self-regulation
SS: Sensory sensitivity
ST: Stereotypy
TC: Tracking capacity
TD: Typically developed
TOT: Total score
UB: Unusual behaviors
