# Supplementary material for: Improved emotion differentiation under reduced acoustic variability of speech in autism
Source: BMC Med. 2024 Mar 14;22:121. doi: 10.1186/s12916-024-03341-y (PMC10941423; doi:10.1186/s12916-024-03341-y)
Supplement: Supplementary file 1 — Additional file 1. An additional file is available that contains (1) statistical results from pairwise comparisons after non-significant frequentist analysis where the alternative hypothesis was favored by bayes factors (2) statistical results from the sensitivity analysis to control age and gender differences between ASD and TD. A PDF file is provided that includes the following tables. Table S1. Statistical outputs for post-hoc simple simple pairwise comparisons for level 1 in ASD. Code for bayes factor: Anecdotal: “.”, moderate: “*”, strong: “**”, very strong: “***”, extreme: “****”. BF01 < 0 highlights data in favor of the alternative hypothesis (bold). Code for Cohen’s d: small: “*”, medium: “**”, large: “***”. Sum(T) is the sum of T-values within the cluster (i.e., the test-statistic). Table S2. Statistical outputs for post-hoc simple simple pairwise comparisons for human voice in TD. Code for bayes factor: Anecdotal: “.”, moderate: “*”, strong: “**”, very strong: “***”, extreme: “****”. BF01 < 0 highlights data in favor of the alternative hypothesis (bold). Code for Cohen’s d: small: “*”, medium: “**”, large: “***”. Sum(T) is the sum of T-values within the cluster (i.e., the test-statistic). Table S3. Statistical outputs for post-hoc simple simple pairwise comparisons for level 1 in TD. Code for bayes factor: Anecdotal: “.”, moderate: “*”, strong: “**”, very strong: “***”, extreme: “****”. BF01 < 0 highlights data in favor of the alternative hypothesis (bold). Code for Cohen’s d: small: “*”, medium: “**”, large: “***”. Sum(T) is the sum of T-values within the cluster (i.e., the test-statistic). Table S4. Statistical outputs for post-hoc simple simple pairwise comparisons for level 2 in TD. Code for bayes factor: Anecdotal: “.”, moderate: “*”, strong: “**”, very strong: “***”, extreme: “****”. BF01 < 0 highlights data in favor of the alternative hypothesis (bold). Code for Cohen’s d: small: “*”, medium: “**”, large: “***”. Sum(T) is the sum of T-values within [file 12916_2024_3341_MOESM1_ESM.pdf]

Emotion Perception: Electroencephalography

Pairwise comparisons after non-significant frequentist analysis where the alternative hypothesis was favored by bayes factors are available in Table S1, Table S2, Table S3 and Table S4 (ASD level 1, TD human, TD level 1, TD level 2, respectively).

ASD Level 1

Table S1. Statistical outputs for post-hoc simple simple pairwise comparisons for level 1 in ASD. Code for bayes factor: Anecdotal: ".", moderate: "*", strong: "**", very strong: "***", extreme: "****". $BF_{01} < 0$ highlights data in favor of the alternative hypothesis (bold). Code for Cohen's *d*: small: "*", medium: "**", large: "***". Sum(T) is the sum of T-values within the cluster (i.e., the test-statistic).

Comparison	Sum(T)	P-Value	*d*	BF_{01}	Time (s)	Frequency (Hz)	Topography
Anger-Disgust	-3457	0.434	-0.29*	1.75.	[0 0.49]	[5 9]	Cz, Pz, CPz, POz
Anger-Fear	294	0.999	0.21*	2.53.	[0.38 0.57]	[7 9]	Pz, CPz, POz
Anger-Happiness	-8760	0.136	-0.51**	**0.60.**	[0.19 0.60]	[4 8]	Cz, Pz, CPz, POz
Anger-Neutral	-1173	0.838	-0.18*	3.34*	[0 0.28]	[6 8]	
Anger-Sadness	-16231	0.041	-0.47*	**0.15***	[0 0.68]	[4 9]	
Disgust-Happiness	-2069	0.705	-0.57**	**0.51.**	[0.38 0.66]	[4 6]	Pz, CPz, POz
Disgust-Sadness	-8597	0.132	-0.28*	1.83.	[0.96 1.42]	[4 11]	Cz, Pz, CPz, POz
Fear-Happiness	-7937	0.155	-0.26*	1.86.	[0.23 0.80]	[4 7]	Pz, CPz, POz
Fear-Neutral	-2888	0.51	-0.30*	4.48*	[0.24 0.51]	[5 8]	Cz, Pz, CPz, POz
Fear-Sadness	-12679	0.09	-0.22*	1.11.	[0 0.73]	[4 11]	
Happiness-Sadness	-4136	0.38	-0.61**	**0.47.**	[0 0.30]	[5 9]	
Neutral-Sadness	-4185	0.417	-0.22*	2.64.	[0.01 0.50]	[9 15]	

TD Human

Table S2. Statistical outputs for post-hoc simple simple pairwise comparisons for human voice in TD. Code for bayes factor: Anecdotal: ".", moderate: "*", strong: "**", very strong: "***", extreme: "****". $BF_{01} < 0$ highlights data in favor of the alternative hypothesis (bold). Code for Cohen's *d*: small: "*", medium: "**", large: "***". Sum(T) is the sum of T-values within the cluster (i.e., the test-statistic).

Comparison	Sum(T)	P-Value	*d*	BF_{01}	Time (s)	Frequency (Hz)	Topography
Anger-Disgust	-888	0.990	-0.44*	**0.35***	[0.25 0.92]	[10 12]	Cz, Pz, CPz, POz
Anger-Fear	-5657	0.206	-0.27*	1.24.	[0 0.71]	[8 12]	
	-7894	0.100	-0.49*	**0.25***	[0.71 1.14]	[9 14]	
Anger-Happiness	-5967	0.200	-0.37*	**0.66.**	[0.47 1.42]	[9 15]	
	3241	0.504	0.54**	**0.33***	[0.58 1.45]	[38 42]	
Anger-Neutral	-4353	0.300	-0.56**	**0.78.**	[0.84 1.45]	[9 13]	Pz, CPz, POz
	560	0.983	0.40*	1.27.	[0.31 0.64]	[38 41]	Cz, Pz, CPz, POz
	1119	0.89	0.21*	2.68.	[0.60 0.93]	[8 10]	Cz, Pz, CPz
	560	0.98	0.87***	**0.01*****	[0.38 0.64]	[38 41]	Cz, Pz, CPz, POz
Anger-Sadness	-848	0.99	-0.29*	1.23.	[0.65 0.82]	[6 7]	Cz, Pz, CPz

	4141	0.34	0.68**	**0.06****	[0.59 1.45]	[37 42]	Cz, Pz, CPz, POz
Disgust-Happiness	-647	0.97	-0.32*	**0.99.**	[0 0.14]	[7 9]	Fz, AFz
	-841	0.94	-0.008*	5.85*	[0.18 0.31]	[6 8]	
	2707	0.53	0.63**	**0.91.**	[0 1.17]	[35 42]	Cz, Pz, CPz, POz
Disgust-Neutral	1566	0.77	0.50**	**0.59.**	[0.11 0.64]	[34 41]	
	2547	0.54	0.23*	2.35.	[0.23 0.84]	[8 11]	
Disgust-Sadness	-4618	0.26	-0.61**	**0.19***	[0.04 0.38]	[5 10]	Cz, Pz, CPz
	-5155	0.23	-0.30*	3.41*	[0 0.40]	[6 10]	Fz, AFz
	1508	0.81	0.56**	**0.37.**	[0.62 1.07]	[35 41]	Cz, Pz, CPz, POz
	5731	0.20	0.17*	3.41*	[0.79 1.18]	[5 10]	Pz, CPz, POz
Fear-Happiness	645	0.99	0.51**	**0.98.**	[0.91 1.28]	[39 42]	Cz, Pz, CPz, POz
Fear-Neutral	7302	0.13	0.33*	**0.76.**	[0.22 0.92]	[8 11]	
Fear-Sadness	-1677	0.87	-0.14*	3.99*	[0 0.23]	[7 9]	Fz, AFz
	2312	0.72	0.56*	**0.65.**	[0.85 1.14]	[8 12]	Pz, CPz, POz
Happiness-Neutral	3437	0.39	0.25*	2.66.	[0.58 0.96]	[8 12]	Cz, Pz, CPz
Happiness-Sadness	-1301	0.95	-0.23*	2.90.	[0.17 0.39]	[6 9]	
	2743	0.69	0.25*	2.27.	[0.82 1.10]	[6 10]	Pz, POz
Neutral-Sadness	-275	0.99	-0.27*	1.51.	[0.03 0.18]	[7 8]	Fz, AFz
	-1609	0.68	-0.26*	2.15.	[0.51 0.89]	[8 11]	Cz, Pz, CPz
	-2327	0.52	-0.005*	4.11*	[0.14 0.51]	[8 10]	
	618	0.96	0.22*	2.56.	[0.63 0.92]	[37 42]	Pz, CPz, POz
	1703	0.66	0.026*	5.79*	[0.88 1.07]	[6 8]	Pz, POz

TD Level 1

Table S3. Statistical outputs for post-hoc simple simple pairwise comparisons for level 1 in TD. Code for bayes factor: Anecdotal: ".", moderate: "*", strong: "**", very strong: "***", extreme: "****". $BF_{01} < 0$ highlights data in favor of the alternative hypothesis (bold). Code for Cohen's *d*: small: "*", medium: "**", large: "***". Sum(T) is the sum of T-values within the cluster (i.e., the test-statistic).

Comparison	Sum(T)	P-Value	*d*	BF_{01}	Time (s)	Frequency (Hz)	Topography
Anger-Disgust	1883	0.79	0.53**	**0.25***	[0 0.19]	[5 6]	Fz, AFz
Anger-Fear	-1364	0.92	-0.02*	5.83*	[0.17 0.39]	[4 5]	
Anger-Happiness	754	0.97	0.04*	5.71*	[0 0.11]	[5 7]	
	1570	0.79	0.49*	**0.43.**	[0.25 0.47]	[6 8]	Cz, Pz, CPz, POz
	4452	0.30	0.29	1.83.	[0.30 0.69]	[5 9]	Fz, AFz
Anger-Neutral	1799	0.77	0.51**	**0.08****	[0.03 0.35]	[5 7]	Fz, AFz
	3734	0.40	0.44*	**0.73.**	[0.35 0.74]	[4 7]	
Anger-Sadness	-522	0.99	-0.19	3.11.	[0.23 0.40]	[4 5]	
	-1882	0.80	-0.74**	**0.10****	[0 0.32]	[4 5]	Cz, Pz, CPz
	2299	0.67	-0.13	3.87*	[0 0.25]	[5 7]	Fz, AFz
Disgust-Fear	-1150	0.96	-0.19*	2.92.	[0.14 0.41]	[5 7]	Fz, AFz
Disgust-Happiness	7552	0.13	0.52**	**0.93.**	[0.14 0.72]	[5 10]	Cz, Pz, CPz, POz
Disgust-Neutral	-932	0.93	-0.26*	1.72.	[0 0.15]	[5 7]	
	5270	0.22	0.67**	**0.09****	[0.18 0.64]	[4 6]	Fz, AFz
Disgust-Sadness	-5884	0.22	-0.19*	3.10*	[0 0.25]	[4 7]	Cz, Pz, CPz, POz
Fear-Happiness	1693	0.75	0.18*	3.32*	[0.32 0.56]	[5 7]	Fz, AFz
Fear-Neutral	9371	0.08	0.75**	**0.002******	[0.11 0.74]	[4 7]	

Fear-Sadness	-3149	0.50	-0.02*	5.80*	[0 0.22]	[4 6]	Cz, Pz, CPz
	873	0.97	0.04*	5.69*	[0.05 0.27]	[5 7]	Fz, AFz
Happiness-Neutral	2657	0.51	0.67**	**0.03*** **	[0.22 0.59]	[4 5]	
Happiness-Sadness	-1526	0.78	-0.21	2.56.	[0.03 0.22]	[4 5]	Cz, Pz, CPz
	-11208	0.05	-0.64**	**0.32***	[0.16 0.74]	[6 9]	Cz, Pz, CPz, POz
Neutral-Sadness	-329	0.99	-0.48*	4.64*	[0.46 0.57]	[5 6]	Pz, POz
	-4434	0.31	-0.87***	**5.75.e-4******	[0 0.40]	[4 5]	Cz, Pz, CPz
	-8011	0.10	-0.64**	**0.02*** **	[0.07 0.67]	[4 6]	Fz, AFz

TD Level 2

Table S4. Statistical outputs for post-hoc simple simple pairwise comparisons for level 2 in TD. Code for bayes factor: Anecdotal: ".", moderate: "*", strong: "**", very strong: "***", extreme: "****". $BF_{01} < 0$ highlights data in favor of the alternative hypothesis (bold). Code for Cohen's *d*: small: "*", medium: "**", large: "***". Sum(T) is the sum of T-values within the cluster (i.e., the test-statistic).

**Comparison**	**Sum(T)**	**P-Value**	***d***	**BF$_{01}$**	**Time (s)**	**Frequency (Hz)**	**Topography**
Anger-Disgust	1277	0.94	0.23*	2.36.	[0.41 0.77]	[19 20]	Cz, Pz, CPz, POz
	936	0.99	0.51**	**0.41.**	[0.11 0.39]	[9 11]	Fz, AFz
Anger-Fear	-448		-0.40*	**0.36.**	[0.81 0.97]	[18 20]	Cz, Pz, CPz
	621		0.57**	**0.03*** **	[0.26 0.44]	[12 13]	
	493		0.47*	**0.55.**	[0.08 0.25]	[11 14]	
	1753	0.80	0.56**	**0.03*** **	[0.18 0.42]	[11 14]	Fz, AFz
	3962	0.37	0.14*	4.00*	[0.06 0.81]	[19 23]	Cz, Pz, CPz, POz
Anger-Happiness	3552	0.45	0.21*	2.88.	[0.27 0.84]	[13 17]	Cz, Pz, CPz
Anger-Neutral	-5528	0.25	-0.55**	**0.18***	[0.80 1.43]	[16 21]	Cz, Pz, CPz, POz
	1683	0.82	0.32*	2.00.	[0.07 0.42]	[10 14]	Cz, Pz, CPz
Anger-Sadness	691	0.99	0.53**	**0.42.**	[1.01 1.40]	[19 22]	Cz, Pz, CPz, POz
	9527	0.06	0.61**	**0.01*** **	[0 0.41]	[9 16]	
	9141	0.07	0.59**	**0.04** **	[0 0.57]	[8 13]	Fz, AFz
Disgust-Fear	842	0.99	0.18*	3.23*	[0.82 1.01]	[16 19]	Cz, Pz, CPz
	1166	0.95	0.19*	3.00*	[0.19 0.62]	[19 22]	Pz, CPz, POz
Disgust-Happiness	-461	0.99	-0.28*	1.43.	[0.43 0.62]	[21 23]	
Disgust-Neutral	-6168	0.22	-0.53**	**0.27***	[0.38 1.28]	[16 23]	Cz, Pz, CPz, POz
	1964	0.80	0.02*	5.83*	[1.21 1.45]	[10 13]	Pz, POz
Disgust-Sadness	-2597	0.62	-0.04*	5.06*	[0.34 0.78]	[21 24]	Cz, Pz, CPz, POz
	1629	0.85	0.30*	1.78.	[0.73 1.06]	[12 16]	Cz, Pz, CPz
	3200	0.49	0.26*	1.87.	[0 0.38]	[9 14]	Cz, Pz, CPz, POz
	2829	0.59	0.11	4.08*	[0.09 0.49]	[9 13]	Fz, AFz
Fear-Happiness	-4092	0.36	-0.48*	**0.83.**	[0.17 0.79]	[18 23]	Cz, Pz, CPz, POz
	-4874	0.29	-0.19*	3.17*	[0 0.60]	[10 15]	Fz, AFz
	1467	0.88	0.31*	1.45.	[0 0.30]	[22 28]	Cz, Pz, CPz, POz
Fear-Neutral	-3158	0.54	-0.56**	**0.05** **	[0.38 1.03]	[19 25]	

	-3165	0.54	-0.65**	**0.03*** **	[0.17 0.62]	[17 22]	
	-6585	0.16	-0.49*	**0.10** **	[0.82 1.45]	[15 25]	
Fear-Sadness	-4575	0.33	-0.63**	**0.02*** **	[0.16 0.78]	[17 25]	
	2268	0.73	0.05*	4.94*	[0 0.26]	[11 15]	
Happiness-Neutral	-4393	0.35	-0.41*	**0.50.**	[0 0.67]	[22 28]	
	-7993	0.12	-0.75**	**0.02*** **	[0.75 1.20]	[14 26]	
	1069	0.96	0.33*	**0.86.**	[0.11 0.43]	[10 12]	Fz, AFz
Happiness-Sadness	319	0.99	0.48*	**0.11***	[0.15 0.34]	[12 14]	Pz, POz
	5045	0.27	0.51**	**0.22***	[0 0.46]	[9 13]	Cz, Pz, CPz, POz
	13024	0.02	0.64**	**0.83.**	[0 0.59]	[8 15]	Fz, AFz
Neutral-Sadness	636	0.99	0.13*	4.37*	[0 0.23]	[12 14]	Cz, Pz, CPz, POz
	691	0.99	0.24*	2.12.	[0 0.10]	[9 11]	Pz, CPz, POz
	876	0.98	0.02*	5.29*	[0.47 0.33]	[12 14]	Fz, AFz
	2215	0.72	0.29*	1.32.	[0.07 0.44]	[9 11]	
	12289	0.04	0.58**	**0.05** **	[0.65 1.23]	[7 22]	Cz, Pz, CPz, POz

Sensitivity analysis: age- and gender-controlled sample

Selection of age- and gender-controlled sample

To control both age and gender, only boys were selected from the original sample. Therefore, 35 ASD and 18 TD boys were pre-selected for the age- and gender-controlled subsample. Then, a selection of children based on propensity score matching considering the age variable was achieved. R-4.3.1 was used. The propensity score was defined as the probability of each child to belong to the ASD group based on age. Particularly, a logistic regression model was built, using a Maximum Likelihood Estimator to optimize the coefficients. Outputs from logistic regression were as follows: estimate = 0.56, standard error = 0.19, z-value = 2.90, p-value = 0.004, residual deviance = 57.54, log likelihood = -28.77, Akaike Inferior Criteria = 61.54, odd ratio = 1.75. The residual variance was computed to confirm the absence of overdispersion ($^{residual\ deviance}/_{degree\ of\ freedom} = 1.13$). Then, samples from both groups (ASD and TD) were matched based on propensity scores (samples from TD were matched to samples from ASD with similar propensity scores). An optimal pair matching without replacement (once an ASD sample was matched, it was not available for matching anymore) was used that collectively minimizes the sum of the absolute pair distances within the matched sample. Therefore, 18 ASD and 18 TD children were selected after the matching process (17 unmatched ASD).

Particularly, 18 children per group were selected for which propensity scores lied in range [12.3% - 79.6%]. Resulting values for matching assessment variables are presented in Table S5. In this table, “Std. Mean. Diff.” is the standardized mean difference of age between ASD and TD. “Var. Ratio” is the ratio of the variance of age in ASD to that in TD. “Mean eCDF” and “Max eCDF” are respectively the average and

maximum distance between the empirical cumulative distribution function of the age variable across groups.

Table S5. Balance assessment before and after matching considering the "age" variable on the boy-only subsample.

**Variable**	**Means TD**	**Means ASD**	**Std. Mean. Diff**	**Var. Ratio**	**Mean eCDF**	**Max eCDF**
Age – pre-selected subsample	10.76	9.09	1.21	0.58	0.29	0.58
Age – Age- and gender-controlled subsample	10.76	10.40	0.26	0.76	0.10	0.33
% balance improvement	N/A	N/A	78.50	48.80	65.50	42.10

To confirm no difference of age between ASD and TD, a two-sample t-test was applied (t = 0.73, p-value = 0.47, d = -0.24). Normality was confirmed by Shapiro-Wilk (W_{TD}=0.98, p-value$_{TD}$=0.97; W_{ASD}=0.90, p-value$_{ASD}$=0.06). The absence of skewness and outliers was outlined. Equality of variances was confirmed by an F-test (F = 0.76, p-value = 0.57). A Bayesian two-sample t-test outlined evidence towards the null hypothesis (BF_{01} = 2.53).

Mixed within-between 2-way ANOVA on Tracking Capacity (TC)

A mixed within-between two-way ANOVA was conducted using R-4.3.1 to assess the effects of group (ASD versus TD), voice (human, level 1, and level 2), and of the interaction between factors on TC scores (*m*): Group x Voice with "Voice" as the repeated (within) factor and "Group" as the independent (between) factor. Normality of residuals was confirmed by Shapiro-Wilk test (W = 0.99, p-value = 0.38). A violation of sphericity was highlighted by Mauchly's test (W_{voice}= 0.80, p-value$_{voice}$ = 0.02; $W_{interaction}$= 0.80, p-value$_{interaction}$ = 0.02). Therefore, a Greenhouse-Geisser correction was performed ($F_{GGe\text{-}voice}$ = 0.83, p-value$_{voice}$ = 0.36, $F_{GGe\text{-}interaction}$ = 0.83, p-value$_{voice}$ = 0.47). Outputs for the group effect (between) were as follows: F(1,34) = 18.00, p-value < 0.001, η^2_G = 0.32. A Bayesian within-between two-way ANOVA outlined evidence towards the null hypothesis for the voice effect (BF_{01} = 5.13, moderate), but highlighted evidence towards the alternative hypothesis for the Voice + Group + Voice*Group model (BF_{01} = 0.22, moderate). Post-hoc analysis correcting for multiple testing revealed evidence for the null hypothesis to all pairwise comparisons within the voice factor for both TD and ASD, and evidence for the alternative hypothesis for ASD versus TD comparisons. Following the same methodology, the mixed within-between two-way ANOVA was conducted without outliers to evaluate the influence of those values on statistical significance. The model was robust to influential values.

Mixed within-between 3-way ANOVA on Event-Related Spectral Perturbations (ERSP)

A mixed within-between three-way ANOVA was conducted to assess the effects of group (ASD versus TD), emotion (Anger, Disgust, Fear, Happiness, Neutral, Sadness), and voice (human, level 1, and level 2) on ERSP. Specifically, a Group x Emotion x Voice ANOVA with "Emotion" and "Voice" as the repeated (within) factors and "Group" as the independent (between) factor was implemented. The interactions between factors were also evaluated: (a) effect of the interaction between group and emotion, (b) effect of the interaction between group and voice, (c) effect of the interaction between emotion and voice, and (d) effect of the three-way interaction between group, emotion, and voice. A non-parametric cluster-based permutation approach was conducted, using Fieldtrip Toolbox from Matlab, so that statistical effects could be related to channel x frequency x time windows (i.e., clusters). Significant statistical outputs for main effects of group, emotion, and interactions are detailed in Table S6. Non-significant clusters of channel x frequency x time windows of interest are also detailed in Table S6.

Table S6. Statistical outputs for main effects of group, emotion, voice, and interactions on the age- and gender-controlled sample. Code for bayes factor: Anecdotal: ".", moderate: "*", strong: "**", very strong: "***", extreme: "****". $BF_{01} < 0$ highlights data in favor of the alternative hypothesis (bold). Sum(F) is the sum of F-values within the cluster (i.e., the test-statistic). Degrees of freedom of the F value are also indicated (factor: numerator, residuals: denominator).

Effect	Sum(F)	P-Value	BF_{01}	Time (s)	Frequency (Hz)	Topography
Group F(1,34)	-28867.51	0.017	N/A	[0 0.43]	[6.01 43.63]	Cz, Pz, CPz, POz
	-10363.62	0.129	0.43.	[0 0.48]	[8.38 21.98]	Fz, AFz
Emotion F(5,170)	16653.60	0.092	4.21*	[0.81 1.45]	[5.54 11.00]	Cz, Pz, CPz, POz
	1454.80	0.999	**23.66****	[0.31 0.83]	[45.44 52.05]	Fz, AFz
Voice F(2,68)	28662.61	0.080	0.50.	[0 1.13]	[11.22 27.87]	Cz, Pz, CPz, POz
	105883.51	0.457	**0.23***	[0 0.54]	[17.10 29.83]	Fz, AFz
	5866.8	0.812	**0.23***	[0.30 0.52]	[4.97 8.85]	Cz, Pz, CPz, POz
Group*Emotion F(5,170)	-36389.39	0.022	N/A	[0 1.45]	[10.70 25.35]	
	15471.43	0.069	1.54.	[0.08 1.15]	[4 8.56]	
Group*Voice F(2,68)	33762.67	0.012	N/A	[0 0.30]	[4.90 44.83]	
	13745.12	0.066	**0.02*****	[0 0.28]	[10.49 36.82]	Fz, AFz
	5566.43	0.283	**0.58.**	[0 0.24]	[45.75 69.22]	Cz, Pz, CPz, POz
	1342.99	0.848	**0.28***	[0 0.25]	[38.61 48.30]	Fz, AFz
	830.98	0.932	8.96*	[0 0.11]	[6.75 8.97]	
Emotion*Voice F(10,340)	142921.18	0.010	N/A	[0 1.45]	[4 24.34]	Cz, Pz, CPz, POz
	74496.61	0.033	N/A	[0.27 1.43]	[4 10.63]	Fz, AFz
	38339.70	0.084	37.13***	[0.26 1.45]	[22.43 44.83]	Cz, Pz, CPz, POz
	35059.60	0.092	3.01*	[0 1.45]	[17.21 30.44]	Fz, AFz
	33789.17	0.097	87.17***		[50.66 69.69]	Cz, Pz, CPz, POz
	7535.45	0.651	8.32*	[0 0.82]	[52.76 64.24]	Fz, AFz
	3185.38	0.932	102.81****	[0.85 1.45]	[79.23 86.02]	Cz, Pz, CPz, POz
Group*Emotion *Voice F(10,340)	81649.40	0.001	N/A	[0 1.45]	[4 29.63]	Cz, Pz, CPz, POz
	37372.21	0.005	N/A		[31.28 73.58]	
	13510.26	0.052	469.25****	[0.83 1.45]	[4 13.12]	Fz, AFz
	4662.91	0.380	296.60****	[0.64 1.29]	[18.80 26.40]	
	3665.23	0.525	34.88***	[0 1.40]	[36.32 42.75]	

Bayesian models on ERSP averaged within non-significant clusters were conducted using JASP to assess the evidence towards the null hypothesis (BF_{01}). Priors were uniformly distributed for ANOVA, and a Cauchy distribution was implemented for pairwise comparisons. Normality of residuals (ANOVA) and of differences (pairwise comparisons) were evaluated by Q-Q plots (observed quantiles as regards theoretical ones) and by Shapiro-Wilk's test. Models were not robust to outliers as normality was not met in their presence, and outputs had different interpretations. Therefore, results from models that did not include outliers were heeded. Furthermore, sphericity was validated by Mauchly's test ($p\text{-value} > 0.05$) for all within-subject effects. BF_{01} are presented in Table S6.

Overall, the sensitivity analysis highlighted the robustness of statistical analysis after controlling age and gender between groups. Slight differences on channel x frequency x time windows of ERSP clusters may have been triggered by the lower statistical power induced by lower sample size.
